# AtSWEET11 and AtSWEET12 transporters function in tandem to modulate sugar flux in plants

**DOI:** 10.1002/pld3.481

**Published:** 2023-03-08

**Authors:** Urooj Fatima, D. Balasubramaniam, Wajahat Ali Khan, Manu Kandpal, Jyothilakshmi Vadassery, Arulandu Arockiasamy, Muthappa Senthil‐Kumar

**Affiliations:** ^1^ National Institute of Plant Genome Research New Delhi India; ^2^ Membrane Protein Biology Group International Centre for Genetic Engineering and Biotechnology New Delhi India

**Keywords:** abiotic stress, biotic stress, pathogen infection, sugar flux, sugar transporters

## Abstract

The sugar will eventually be exported transporter (SWEET) members in Arabidopsis, AtSWEET11 and AtSWEET12 are the important sucrose efflux transporters that act synergistically to perform distinct physiological roles. These two transporters are involved in apoplasmic phloem loading, seed filling, and sugar level alteration at the site of pathogen infection. Here, we performed the structural analysis of the sucrose binding pocket of AtSWEET11 and AtSWEET12 using molecular docking followed by rigorous molecular dynamics (MD) simulations. We observed that the sucrose molecule binds inside the central cavity and in the middle of the transmembrane (TM) region of AtSWEET11 and AtSWEET12, that allows the alternate access to the sucrose molecule from either side of the membrane during transport. Both AtSWEET11 and AtSWEET12, shares the similar amino acid residues that interact with sucrose molecule. Further, to achieve more insights on the role of these two transporters in other plant species, we did the phylogenetic and the *in‐silico* analyses of *AtSWEET11* and *AtSWEET12* orthologs from 39 economically important plants. We reported the extensive information on the gene structure, protein domain and *cis*‐acting regulatory elements of *AtSWEET11* and *AtSWEET12* orthologs from different plants. The cis‐elements analysis indicates the involvement of *AtSWEET11* and *AtSWEET12* orthologs in plant development and also during abiotic and biotic stresses. Both in silico and *in planta* expression analysis indicated *AtSWEET11* and *AtSWEET12* are well‐expressed in the Arabidopsis leaf tissues. However, the orthologs of *AtSWEET11* and *AtSWEET12* showed the differential expression pattern with high or no transcript expression in the leaf tissues of different plants. Overall, these results offer the new insights into the functions and regulation of *AtSWEET11* and *AtSWEET12* orthologs from different plant species. This might be helpful in conducting the future studies to understand the role of these two crucial transporters in Arabidopsis and other crop plants.

## 1 INTRODUCTION

Plant sugar will eventually be exported transporter (SWEET) proteins were initially identified in the model organism *Arabidopsis thaliana* (Chen et al., 2010). In total, 17 members were identified in Arabidopsis, classified into four different sub‐clades (Breia et al., 2021; Chen et al., 2010; Feng & Frommer, 2015). All members of Clade III including AtSWEET11 and AtSWEET12 were characterized as sucrose efflux transporters using forster resonance energy transfer (FRET) based sucrose sensors expressed in human embryonic kidney (HEK293T) cells, yeasts, and time‐dependent efflux of [^14^C] sucrose in *Xenopus* oocytes (Chen et al., 2012). AtSWEETs are bidirectional transporters that facilitate the diffusion of sucrose molecules down the concentration gradient and adapt a uniporter transport mechanism as their transport activity is pH independent. Kinetic studies of AtSWEET12 (Km for sucrose uptake and efflux was ~70 mM and 10 mM respectively) revealed that SWEETs are low affinity sucrose transporters (Chen et al., 2012). AtSWEET11 and AtSWEET12 share almost 88% amino acid similarity (Chen et al., 2012) and both were shown to exhibit the substrate flexibility as they transported glucose and fructose in addition to sucrose (Le Hir et al., 2015). These SWEET proteins are heptahelical transmembrane (TM) transporters with two internal parallel triple‐helix bundles (THB) that are interconnected by the nonconserved helix TM4 (Han et al., 2017; Tao et al., 2015). The two THB domains have a twofold rotation symmetry perpendicular to the membrane plane and have a characteristic 1–3‐2 and 5–7‐6 topological arrangement (Anjali et al., 2020; Han et al., 2017).

Among the members of *AtSWEETs*, *AtSWEET11* and *AtSWEET12* are particularly known to be expressed in all tissues, including the leaves, roots, seeds, siliques, and flowers (Chen et al., 2012). In Arabidopsis, AtSWEET11 and AtSWEET12 participate in pivotal processes such as phloem loading (Chen et al., 2012), xylem development (Le Hir et al., 2015), and seed filling (Chen et al., 2015). This demonstrates that AtSWEET11 and AtSWEET12 are crucial transporters required in major developmental and physiological processes. Besides, these two transporters optimize the sugar flux in response to varying environmental conditions. AtSWEET11 and AtSWEET12 have been shown to have role during interactions with pathogens (Chen et al., 2010; Gebauer et al., 2017; Walerowski et al., 2018). Nevertheless, the dynamic role of these two transporters in multiple integrated aspects of plant physiology has raised considerable interest in their structure and molecular regulatory mechanisms. In this article, we probe the structural aspects of sucrose binding pockets from AtSWEET11 and AtSWEET12 with the help of molecular docking and molecular dynamics simulations. Further, we conducted the detailed analysis of *AtSWEET11* and *AtSWEET12* orthologs from 20 different plant families covering almost 39 economically important plants, to deduce the information on the gene structure, protein domain, *cis*‐acting regulatory elements and the post‐translational modification sites that offers the critical insights about the functions and regulation of *AtSWEET11* and *AtSWEET12* orthologs from other plant species.

## 2 MATERIALS AND METHODS

### 2.1 Plant growth

Arabidopsis seeds of Columbia‐0 (Col‐0); T‐DNA mutants of *AtSWEET11* and *AtSWEET12* genes (SALK_073269C and SALK_031696C) were sown in a soil mixture of 3:1 vol/vol agropeat (Prakruthi Agro Tech, Bangalore, India) and vermiculite (Keltech Energies Ltd., Bangalore, India) and then cold treated for 3 days at 4 °C in the dark. Arabidopsis plants were grown in a growth chamber (PGR15; Conviron, Winnipeg, Canada) under 8 h light (light intensity, 200 μE m^−2^ s^−1^)/16 h dark at 20°C and 75% relative humidity. Plants were irrigated alternately with water and 1/2X Hoagland nutrient solution (Cat# TS1094; HiMedia Laboratories, Mumbai, India) every day.

### 2.2 Bacterial pathogen and plant inoculations

The host bacterial pathogen of Arabidopsis, *Pseudomonas syringae* pv. *tomato* DC3000 (*Pst* DC3000) were grown at 28°C with continuous shaking at 150 rpm in King's B (KB) medium (liquid) (Cat# M1544; HiMedia Laboratories) containing rifampicin at 50 μg/ml. Bacterial cultures were grown overnight (12 h) to obtain an optical density of .4 at 600 nm (OD_600_ = .4). Bacterial cells were collected by centrifugation at 4,270 *× g* for 10 min, washed thrice in sterile water, and re‐suspended in sterile water at desired concentrations. The concentrations used for the inoculation of the leaves (32‐day‐old plants) were 5 × 10^5^ colony‐forming units (CFU)/mL. The 5 ml of bacterial suspension was syringe‐infiltrated on the abaxial surface of fully expanded leaves using a needleless syringe. The inoculated plants were maintained in a growth chamber at 20°C.

### 2.3 Molecular dynamics (MD) trajectory analysis

The full‐length amino acid sequences of AtSWEET11 and AtSWEET12 were obtained from TAIR (https://www.arabidopsis.org) and submitted to psiPRED server (http://bioinf.cs.ucl.ac.uk/psipred) for sequence based secondary structure prediction. Psipred predicted transmembrane helices and cytoplasmic disordered C‐terminal region for both the sequences. For homology modeling, the methodology from Sastry et al. (2013) was followed. The full length AtSWEET11 and AtSWEET12 protein sequence were submitted to the Robetta server (http://robetta.bakerlab.org) and the output models for AtSWEET11 (PD algorithm) and AtSWEET12 (TR algorithm) with .76 and .74 confidence score respectively were selected after visualizing them. The models selected had minimal estimated positional error for the residues. However, the C‐terminal for both the predicted proteins was highly disordered with maximal regions modeled as loops. The 3D coordinates of AtSWEET11 and AtSWEET12 homology models were prepared using the protein preparation wizard of the Schrodinger suite. The missing residues and side chains were filled and bond order errors were corrected, followed by H‐bond optimisation and a restrained minimisation with a cut‐off of .3 Å. The prepared protein models were simulated to equilibrate in a POPC lipid bilayer at 300 K. POPC lipid was automatically placed perpendicular to the transmembrane helices and the systems were solvated using TIP3P water model. The MD system was electrostatically neutralized by placing counter Cl^−^ ions and .15 M KCl was added to provide adequate ionic strength. The prepared systems were relaxed by default desmond relaxation protocols before being simulated for 600 ns with a recording interval of 50 ps. The C‐terminal domain was excluded due to high level of disorder and only the stable transmembrane domain of 1–219 amino acids was used for docking and MD analysis.

### 2.4 RMSD plot analysis

The 3D coordinates of sucrose were extracted from PDB:3LDK and possible conformers were generated using Ligprep. The 3D coordinates of the AtSWEET11 and AtSWEET12 transmembrane domain were extracted from the last frame of the 600 ns MD and subjected to a sitemap analysis. The best scoring sites with site scores of 1.139 and 1.134 were predicted at the central cavity for AtSWEET11 and AtSWEET12 respectively. These sites were selected to generate receptor grids for docking sucrose. The sucrose was docked at the prepared grid using the XP (Extra precision) docking mode using Glide. Output poses were visualized for interactions and clashes, and binding energy calculations were calculated through Prime‐MMGBSA module of Schrodinger suite to select the best pose for MD run. The sucrose docked AtSWEET11 and AtSWEET12 were prepared in lipid bilayer membrane environment and simulated for 500 ns and 1us respectively to study their interaction dynamics. Thermal MM‐GBSA script was ran on the MD trajectory of AtSWEET11 and AtSWEET12 to obtain dG binding score w.r.t. Frames. The most stable complex according to the dG binding score was exported to study interactions/representation.

### 2.5 Multiple sequence alignment

The full‐length amino acid sequences of AtSWEET11, AtSWEET12, AtSWEET13 and OsSWEET2b were obtained from TAIR (https://www.arabidopsis.org). Sequences were aligned using ClustalW (https://www.genome.jp/tools-bin/clustalw).

### 2.6 Identification of AtSWEET11 and AtSWEET12 orthologous proteins

The potential orthologs of *AtSWEET11* and *AtSWEET12* were identified by comparing the protein sequence against the proteomes of 39 economically important plant species from 20 different families. SWEET protein sequences were retrieved from EnsemblPlants database (http://plants.ensembl.org/). The best five hits for the orthologs of each plant species were again compared against the Arabidopsis proteome. The best three hits were then examined for the presence of MtN3_slv domain (IPR018179) using Pfam database (http://pfam.xfam.org/) and Conserved Domain Database (CDD) (http://www.ncbi.nlm.nih.gov/Structure/cdd/wrpsb.cgi) and also checked for the presence of transmembrane helices (TMHs) in protein sequences using TMHMM Server v.2.0 (http://www.cbs.dtu.dk/services/TMHMM/) with default parameters. Finally, the first best hit obtained after passing through these steps was considered as a potential orthologs of *AtSWEET11* and *AtSWEET12* for each plant species.

### 2.7 Time‐tree and phylogenetic analysis

The time‐tree analysis of thirty‐nine different plant species from twenty different families was performed. The species names were taken as input and time‐tree was generated using the MEGA X (www.megasoftware.net). The nodes in the tree indicate the divergence times for different plant species. The phylogenetic analysis of AtSWEET11 and AtSWEET12 orthologs in thirty‐nine different plant species from twenty different families was performed using the RelTime method. Divergence times for all branching points in the topology were calculated using the Maximum Likelihood method and General Time Reversible model. The tree is drawn to scale with branch lengths measured in the relative number of substitutions per site (Kumar et al., 2018; Tamura et al., 2012). The evolutionary analysis was conducted in MEGA X.

### 2.8 Gene structure, chromosome localization and cis‐elements analysis

The gene structure analysis including exon‐intron arrangement were conducted for the orthologs of *AtSWEET11* gene and *AtSWEET12* gene from thirty‐nine different plant species using Gene Structure Display Server 2.0 (http://gsds.gao-lab.org/index.php). The chromosome location of the orthologous genes was identified using EnsemblPlants database (http://plants.ensembl.org/). The cis‐elements were identified in 1.5 kb 5′ upstream promoter regions of AtSWEET11 and AtSWEET12 orthologs from 39 different plant species using PlantCARE (http://bioinformatics.psb.ugent.be/webtools/plantcare/html/).

### 2.9 Protein motif analysis and tertiary structure prediction

The conserved motifs in orthologous proteins for AtSWEET11 and AtSWEET12 were identified using MEME_suite (https://meme-suite.org/meme/). The value of 0 or 1 was used for a specific motif and 20 was set as the upper limit of motifs. The motif length was set at 6–50 amino acids. All motifs were then annotated using InterProScan database (http://www.ebi.ac.uk/Tools/pfa/iprscan/). The tertiary structures of the proteins orthologus to AtSWEET11 and AtSWEET12 were predicted using Robetta (https://robetta.bakerlab.org/).

### 2.10 RT‐qPCR analysis

The total RNA was extracted from the leaf samples using TriZol reagent (Cat# 15596018, Invitrogen, Carlsbad, CA, USA) as per the manufacturer's protocol. RNA quantification was done using a NanoDrop spectrophotometer (ND‐1000; Thermo Fisher, Waltham, MA, USA). RNA (5 μg) was treated with DNase. First‐strand cDNA synthesis was done using DNase‐treated RNA in a reaction volume of 50 μl by a Verso cDNA synthesis kit (Cat# AB1453A, Thermo Scientific) following the manufacturer's protocol. The gene‐specific primers were designed using Primer 3 software (http://bioinfo.ut.ee/primer3-0.4.0/) (Untergasser et al., 2012). For real‐time quantitative PCR (RT‐qPCR), a 10 μl final volume was prepared by adding 1 μl of five‐fold diluted cDNA, gene‐specific primers at 750 nM each, and HotStart‐IT SYBR Green qPCR Master Mix (Cat# 600882, Agilent Technologies, Santa Clara, CA, USA) as per the manufacturer's protocol. RT‐qPCR was performed according to the manufacturer's instructions on an ABI 7900HT PCR system (Applied Biosystems, Foster City, CA, USA). The cycle threshold (Ct) values for *AtACTIN8 (AT1G49240)* expression were used to normalize the expression values of target genes in each sample. The relative expression values for each sample were determined over their respective control using the comparative 2^−ΔΔCt^ method (Livak & Schmittgen, 2001). Three independent biological replicates were used for all RT‐qPCR analyses.

## 3 RESULTS

### 3.1 AtSWEET11 and AtSWEET12 3D structures based on homology modeling

**FIGURE 1:**
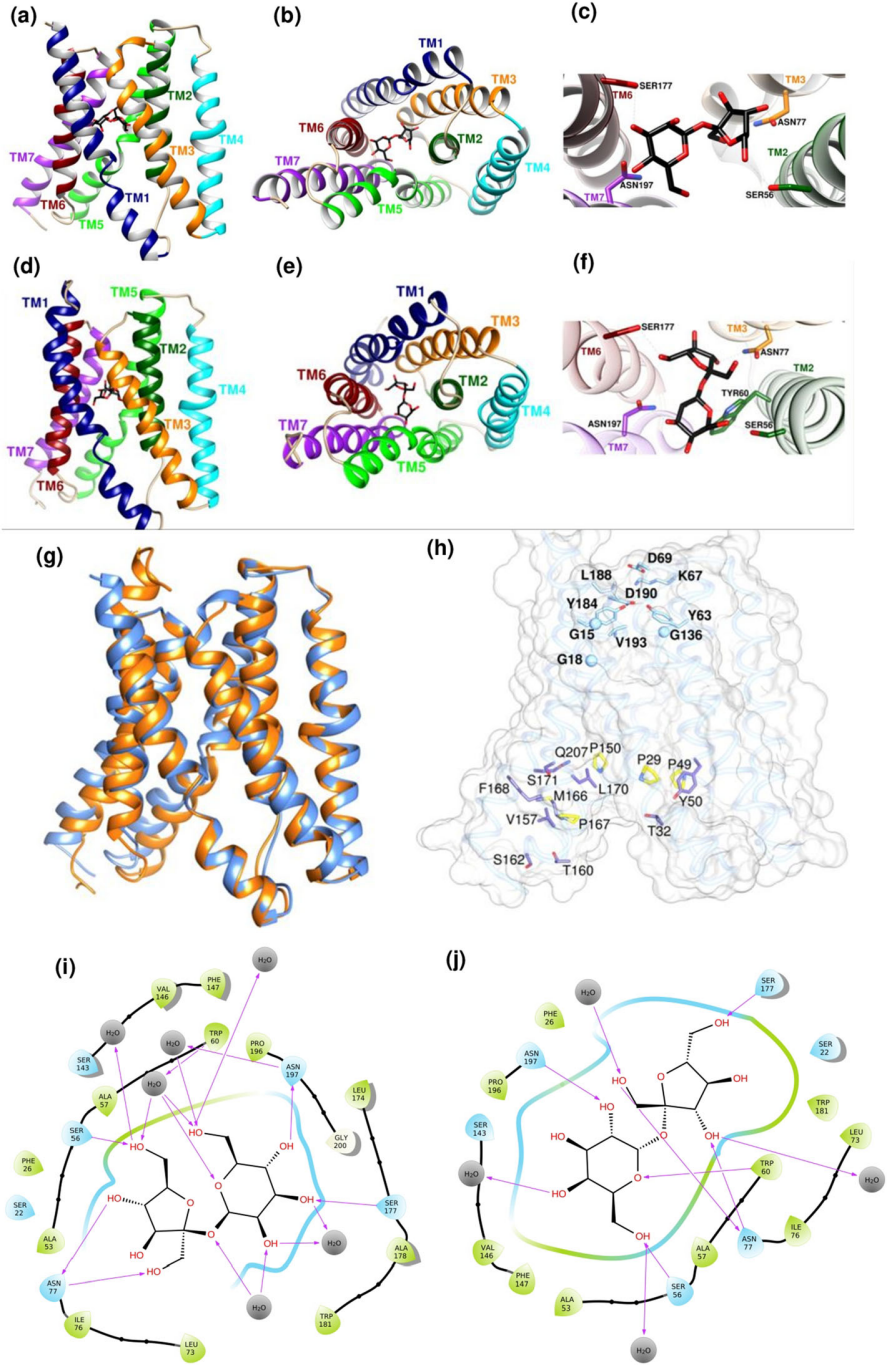
Protein structures of AtSWEET11 and AtSWEET12 and key residues involved in sucrose transport. a–j, in silico homology models of AtSWEET11 and AtSWEET12. The stable docked poses of the SWEET–sucrose complex after molecular dynamics (MD) simulations are shown. The transmembrane (TM) helices are colored (TM1–TM7), and sucrose is represented as a black stick. Ribbon representation of AtSWEET11 in parallel view from the membrane (a), in cytoplasmic view (b), in a close‐up view of sucrose in the central cavity with interacting residues (c). Ribbon representation of AtSWEET12 in parallel view from the membrane (d), in cytoplasmic view (e), in a close‐up view of sucrose in the central cavity with interacting residues (f). (g), structural superposition of AtSWEET11 (cornflower blue) and AtSWEET12 (orange). The root mean square deviation (RMSD) between the structures is 1.48 Å. (h), the residues of the periplasmic gate and cytosolic gate in AtSWEET11 (are represented as a transparent gray surface and blue ribbon), respectively. The residues of the periplasmic gate are shown in cyan. The proline tetrad ring from the cytosolic gate is shown in yellow. The other putative residues that might be involved in cytoplasmic gating during the transport cycle are shown in purple: T32, Y50, V157, T160, S162, M166, F168, L170, S171, and Q207. The sucrose‐interacting diagram for the complex in (i) AtSWEET11 and (j) AtSWEET12. For more details, see Supplementary Figures 1 and 3.

The TM regions of AtSWEET11 and AtSWEET12 were homology modeled using the Robetta server (Yang et al., 2020) (Figure 1 and Supplementary Figure 1) based on the crystal structures of OsSWEET2b (from *Oryza sativa*) (Tao et al., 2015) and AtSWEET13 (Han et al., 2017) have recently been determined. The TM regions of AtSWEET11 and AtSWEET12 share similar structural features with OsSWEET2b and AtSWEET13, as indicated by low root mean square deviation (RMSD) upon superposition (Supplementary Figure 2). Both AtSWEET11 and AtSWEET12 models consist of seven TM helices (TMs 1–7), with N‐terminal THB (TMs 1–3) and C‐terminal THB (TMs 5–7) interconnected by TM4 (Figure 1a, b, d and e). The homology models of AtSWEET11 and AtSWEET12 are in cytoplasmic open conformation (Supplementary Figure 2 and Figure 1g). The TM region for AtSWEET11 and AtSWEET12 from the AlphaFold Protein Structure Database (Jumper et al., 2021; Varadi et al., 2022) were superposed (Cα atoms) with respective models reported in this article and found to be in cytoplasmic open conformation with no significant RMSD. The RMSD plots of the simulated MD runs indicated that the transmembrane region stabilized with an average c‐alpha RMSD of 2.3 Å and 2.06 Å for AtSWEET11 and AtSWEET12 respectively (Supplementary Figure 2). The C‐terminal domain was found to be highly disordered as reflected by high RMSF values and contributed majorly to the overall RMSD of the whole structure throughout the simulation. Hence, the cytoplasmic C‐terminal domain (CTD) of AtSWEET11 (220–289 amino acids) and AtSWEET12 (220–285 amino acid) could not be modeled with accuracy due to lack of homologous structures.

### 3.2 Analysis of AtSWEET11 and AtSWEET12 sucrose‐binding pockets

Molecular docking followed by molecular dynamics (MD) simulations for 500 ns and 1,000 ns, respectively were performed in order to probe the sucrose binding site using site map (Schrödinger's suite) (Supplementary Figures 1 and 3). The MD trajectory analysis for AtSWEET11 and AtSWEET12 sucrose docked poses revealed that the c‐alphas were stable throughout the simulation with an average RMSD of 1.43 Å and 1.93 Å respectively, while RMSD for sucrose was calculated to be 4.42 Å and 2.33 Å respectively (Supplementary Figure 1). For both AtSWEET11 and AtSWEET12, the best scoring sites predicted at the central cavity and in the middle of the TM region (Supplementary Figure 3) were used to dock the sucrose (Figure 1c and f), which is unbiased by the already known crystal structure of AtSWEET13‐dCMP complex. This allows alternate access to the sucrose molecule from either side of the membrane during transport. Interestingly, in both AtSWEET11 and AtSWEET12, the residues S22, S56, W60, N77, N197, W181, S177, V146, and S143 interact with sucrose, with equivalent residues interacting with dCMP in the reported AtSWEET13 structure (Figure 1i and j, Supplementary Table 1). The additional sucrose interacting residues in AtSWEET11 and AtSWEET12 from the molecular simulation is represented as 2D‐Ligand interaction diagram (Figure 1i and j). Among the residues present in the sucrose binding site, Asn77 and Asn197 are highly conserved across all AtSWEETs (except AtSWEET6, wherein Asn77 is replaced by serine) and OsSWEET2b. Multiple sequence alignment of AtSWEET11, AtSWEET12, and AtSWEET13 with OsSWEET2b reveals 61 conserved residues, whereas only 22 are conserved across all the AtSWEETs 1–17. Notably, T159 in AtSWEETs is replaced by serine in OsSWEET2b (Supplementary Figure 4). In both AtSWEET11 and AtSWEET12, Y63, K67, D69, and D190 form the putative extracellular gate (Figure 1h). The Tyr‐Asp pair (Tyr63 and Asp190) that forms the periplasmic gate is highly conserved among SWEETs. However, the interaction of this pair with a Gln132 (OsSWEET2b) or Glu131 (AtSWEET13) is lost due to replacement with Ala in AtSWEET11 and AtSWEET12. Other residues, namely, Y184, V193, and L188, may also form a luminal gate, as mutations in corresponding residues have abolished the transport activity in AtSWEET13 and OsSWEET2b. Of the remaining conserved residues that can form the putative intracellular gate, the proline tetrad (P29 from TM1, P49 from TM2, P150 from TM5, and P167 from TM6) plays a major role in alternating access mechanism of sucrose transport by inducing concerted structural arrangements in the TM helices (Figure 1h). Replacing any one of these prolines with alanine in AtSWEET1 abolishes the transport activity (Tao et al., 2015). When adjacent residues to these conserved prolines were mutated to alanine in AtSWEET1, it resulted in reduced glucose transport (Tao et al., 2015), indicating the importance of these residues in the transport cycle. The other conserved residues that form the putative intracellular gate are shown in Figure 1h. When co‐expressed with a wild type transporter, mutation of V188A in AtSWEET1 in the extrafacial gate showed complete transport inhibition while P23T in the cytosolic gate played an allosteric role, both displaying a dominant‐negative effect in substrate transport (Han et al., 2017; Tao et al., 2015; Xuan et al., 2013).

### 3.3 Analysis of *AtSWEET11* and *AtSWEET12* orthologs from different plant species

**FIGURE 2:**
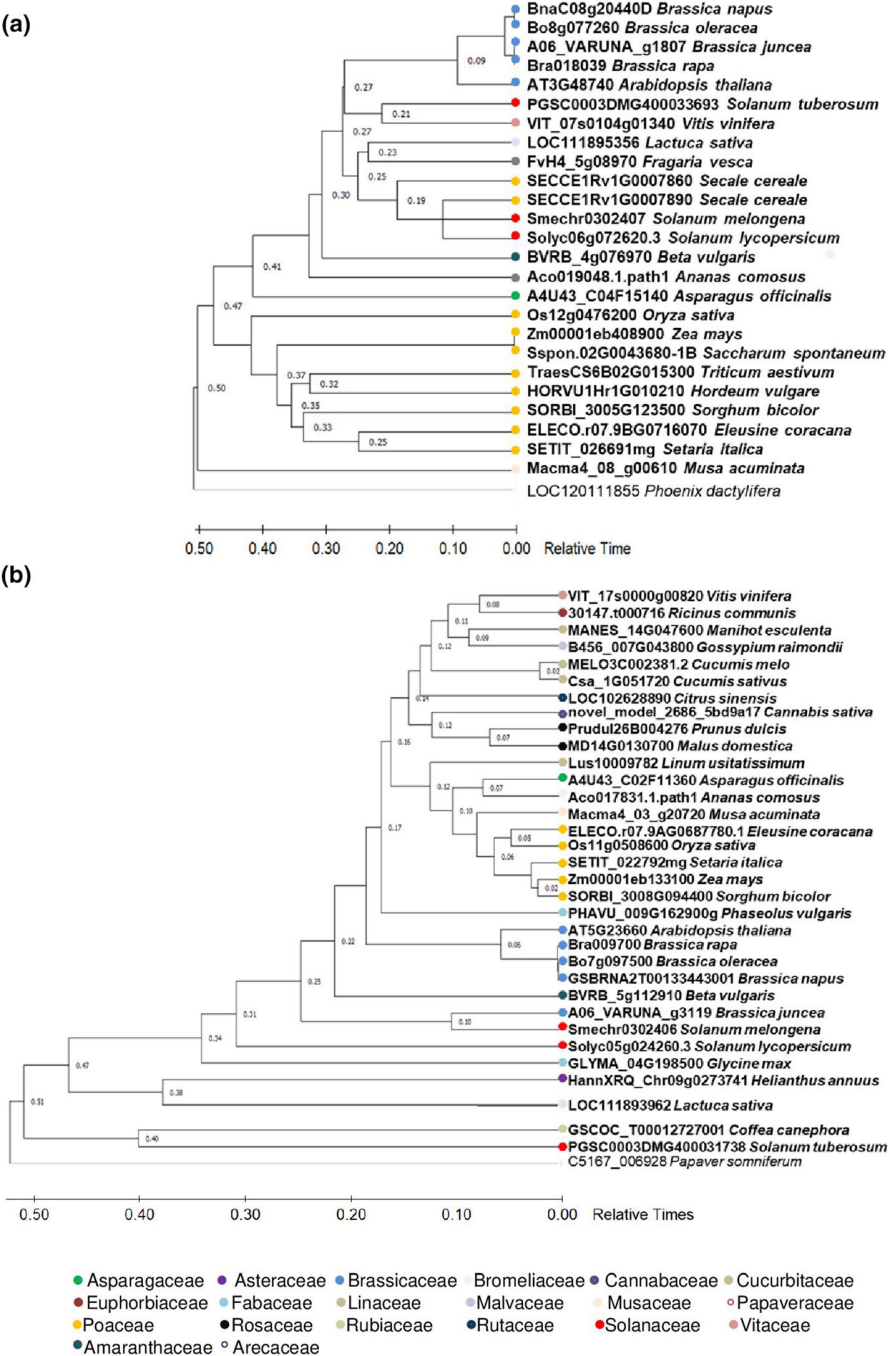
Phylogenetic analysis for AtSWEET11 and AtSWEET12 orthologs in different plant species. The evolutionary analysis of (a), *AtSWEET11* and (b), *AtSWEET12* orthologs in thirty‐nine different plant species from twenty different families was performed. the time‐tree was generated using the RelTime method (1). Divergence times for all branching points in the topology were calculated using the maximum likelihood method and general time reversible model (2). The tree is drawn to scale with branch lengths measured in the relative number of substitutions per site. This analysis involved 25 nucleotide sequences for A, and 33 sequences for B. evolutionary analysis were conducted in MEGA X (3). The name of the families for each species were indicated by colored circle. For more details see Supplementary File 1.

The potential orthologs of *AtSWEET11* and *AtSWEET12* were identified by comparing the protein sequence against the proteomes of 39 economically important plant species from 20 different families (Supplementary File 1a). *AtSWEET11* orthologs were found in 24 of the 39 plant species (Supplementary File 1b), while *AtSWEET12* orthologs were found in 33 plant species (Supplementary File 1c). Interestingly, orthologs exclusive for *AtSWEET11* were found in six different plant species while 12 plant species had orthologs exclusive for *AtSWEET12* (Figure 2 and Supplementary File 1b, c). The absence of either *AtSWEET11* or *AtSWEET12* orthologs in these plant species implies that during the course of evolution, only one transporter might have been responsible for the sugar transport function. Genome sequence analysis of potential orthologous candidates from different plant species indicates the evolutionary and functional significance of these two transporters across the plant kingdom. The time tree analysis of all 39 species demonstrates a distinct bifurcation between monocots and dicots (Figure 2 and Supplementary Figure 5). Similarly, the AtSWEET11 and AtSWEET12 orthologs exhibit conservation within dicot and monocot group, except for *Secale cereal* (Figure 2).

### 3.4 Chromosomal assignment, gene structure and cis‐regulatory elements analysis for *AtSWEET11* and *AtSWEET12* orthologs

**FIGURE 3:**
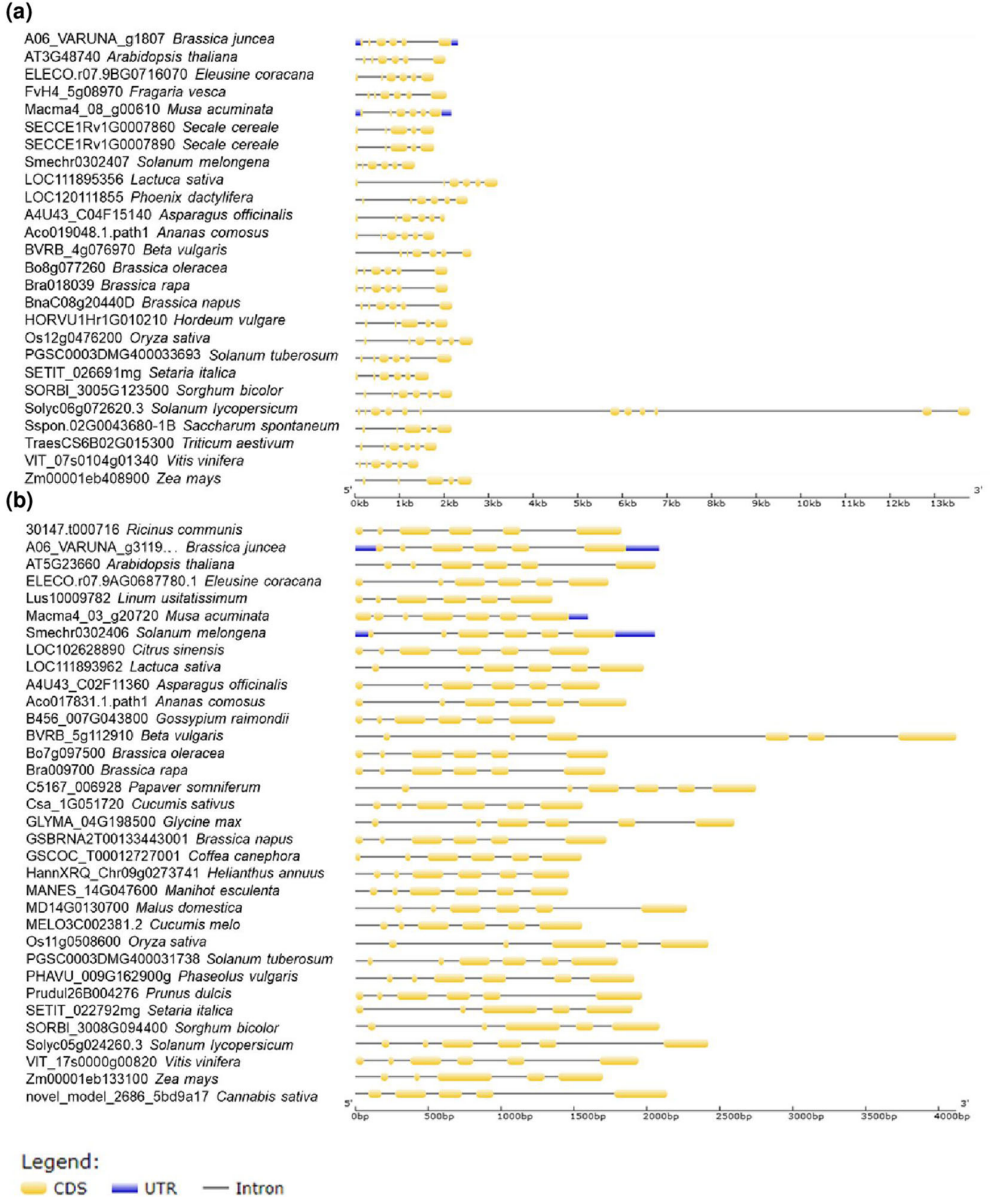
Exon‐intron distribution for *AtSWEET11* and *AtSWEET12* orthologs in different plant species. The gene structure analysis including exon‐intron arrangement were conducted for the orthologs of (a), *AtSWEET11* gene and (b), AtSWEET12 gene from thirty‐nine different plant species using gene structure display server 2.0 (http://gsds.gao‐lab.org/index.php). The analysis involved 25 gene sequences for (a), and 33 gene sequences for (b). exons are represented by yellow color; introns are represented by black line; untranslated‐regions (UTRs) are represented by blue color. For more details see Supplementary File 1.

Chromosomal location and gene structure analysis were performed for the identified orthologs of *AtSWEET11* and *AtSWEET12* (Figure 3 and Supplementary File 1b, c). The gene length for *AtSWEET11* orthologs from different plant species varies from 1,300 to 3,500 bp, with approximately 5–6 exons and 4–6 introns, except in *Solanum lycopersicum*, which has a gene length of approximately 14,000 bp with 12 exons and 12 introns. The orthologs of *AtSWEET12* from different plant species have gene lengths of 1,300–4,000 bp with 6–7 exons and 5–6 introns (Figure 3).

**FIGURE 4:**
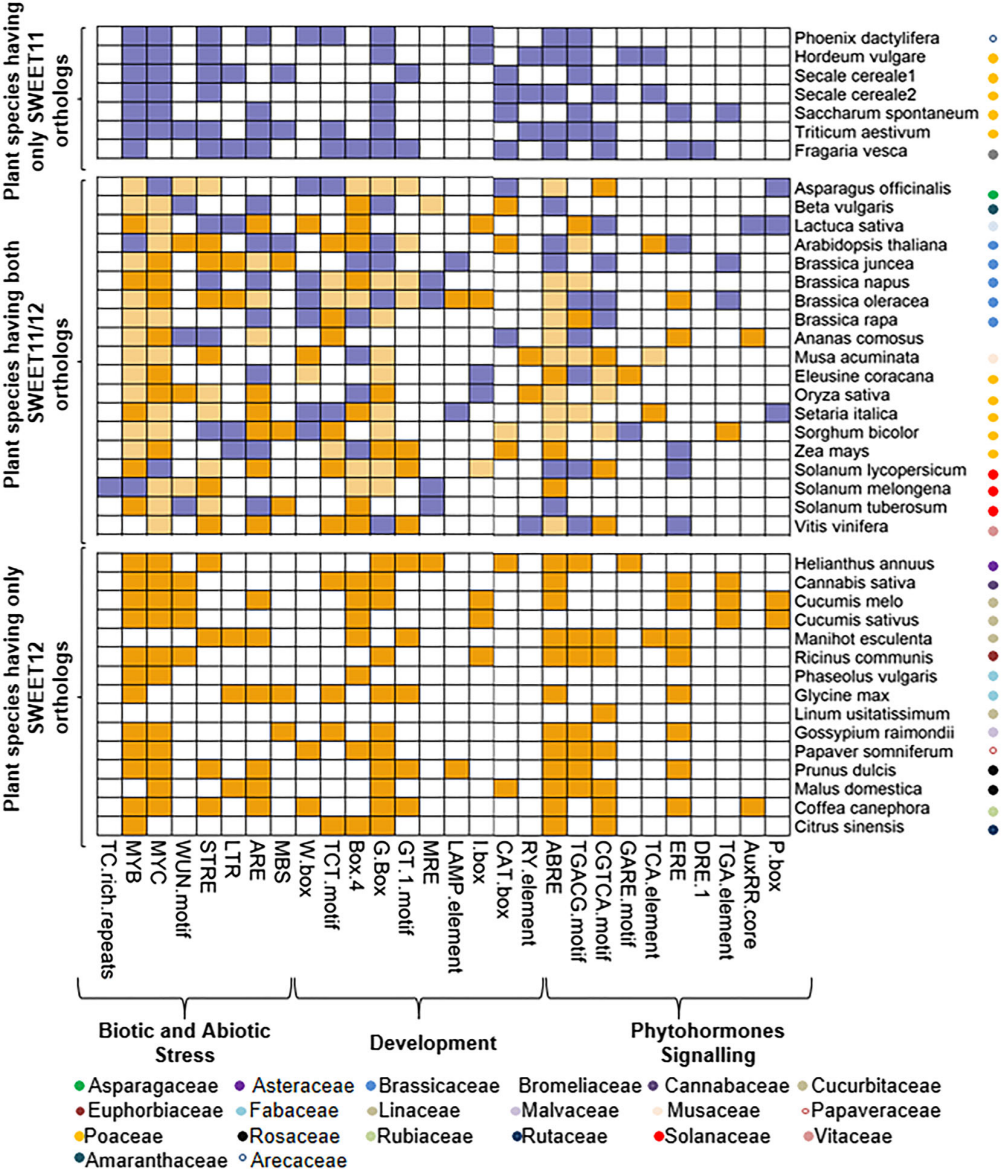
Distribution of *cis*‐acting regulatory elements involved in plant development and stress responses. The *cis*‐elements were identified in 1.5 kb 5′ upstream promoter regions of *AtSWEET11* and *AtSWEET12* orthologs from 39 different plant species using PlantCARE (http://bioinformatics.psb.ugent.be/webtools/plantcare/html/). The analysis involved 25 protein sequences for *AtSWEET11* orthologs and 33 protein sequences for *AtSWEET12* orthologs. The heatmap shows *cis*‐elements identified in *AtSWEET11* orthologs (purple boxes), *cis*‐elements identified in *AtSWEET12* orthologs (orange boxes), *cis*‐elements identified in both *AtSWEET11* and *AtSWEET12* orthologs (light‐orange boxes), and the absence of *cis*‐elements in *AtSWEET11* and *AtSWEET12* orthologs (white boxes) from different plant species. Families of each species are indicated by colored circles. For more details, see Supplementary File 1.

The promoter sequences of *AtSWEET11* and *AtSWEET12* orthologs were analyzed for *cis*‐acting regulatory elements. *Cis*‐elements involved in plant development, including light response, were present in the promoter region of the *AtSWEET11* and *AtSWEET12* orthologs (Figure 4 and Supplementary File 1d, e). G‐box was the most highly distributed *cis*‐element present in the promoter region of the *AtSWEET11* and *AtSWEET12* orthologs from many plant species, followed by Box 4, TCT motif, GT1 motif, LAMP elements, and others. RY *cis*‐elements involved in seed development were present in the promoters of a few *AtSWEET11* and *AtSWEET12* orthologs from Poaceae and a few other families (Figure 4). The *cis*‐elements associated with phytohormone signaling were also present in the promoter sequences of *AtSWEET11* and *AtSWEET12* orthologs. The ABRE, TGACG, and CGTCA elements were highly distributed in the promoter sequences of *AtSWEET11* and *AtSWEET12* orthologs, followed by ERE, TGA, TCA, GARE elements, and others. The DRE1 element was only present in the promoter sequences of *AtSWEET12* orthologs from *Fragaria vesca* (Figure 4). Abiotic and biotic stress‐associated *cis*‐elements involved in responses to heat, cold, drought, wounding, and pathogens were also present in the promoter sequences of *AtSWEET11* and *AtSWEET12* orthologs. MYB and MYC elements were abundantly distributed in the promoter sequences of *AtSWEET11* and *AtSWEET12* orthologs from different plant species, followed by STRE, ARE, LTR, W box, WUN, and MBS elements. Defense and stress response‐related TC‐rich elements were only present in the promoter sequence of the *AtSWEET11* ortholog from *Solanum melongena* (Figure 4). Overall, *cis*‐element analysis of *AtSWEET11* and *AtSWEET12* orthologs indicates that the orthologs of these transporters might play crucial roles in other plant species.

### 3.5 Protein structure, motif and post‐translational modification (PTM) sites analysis

**FIGURE 5:**
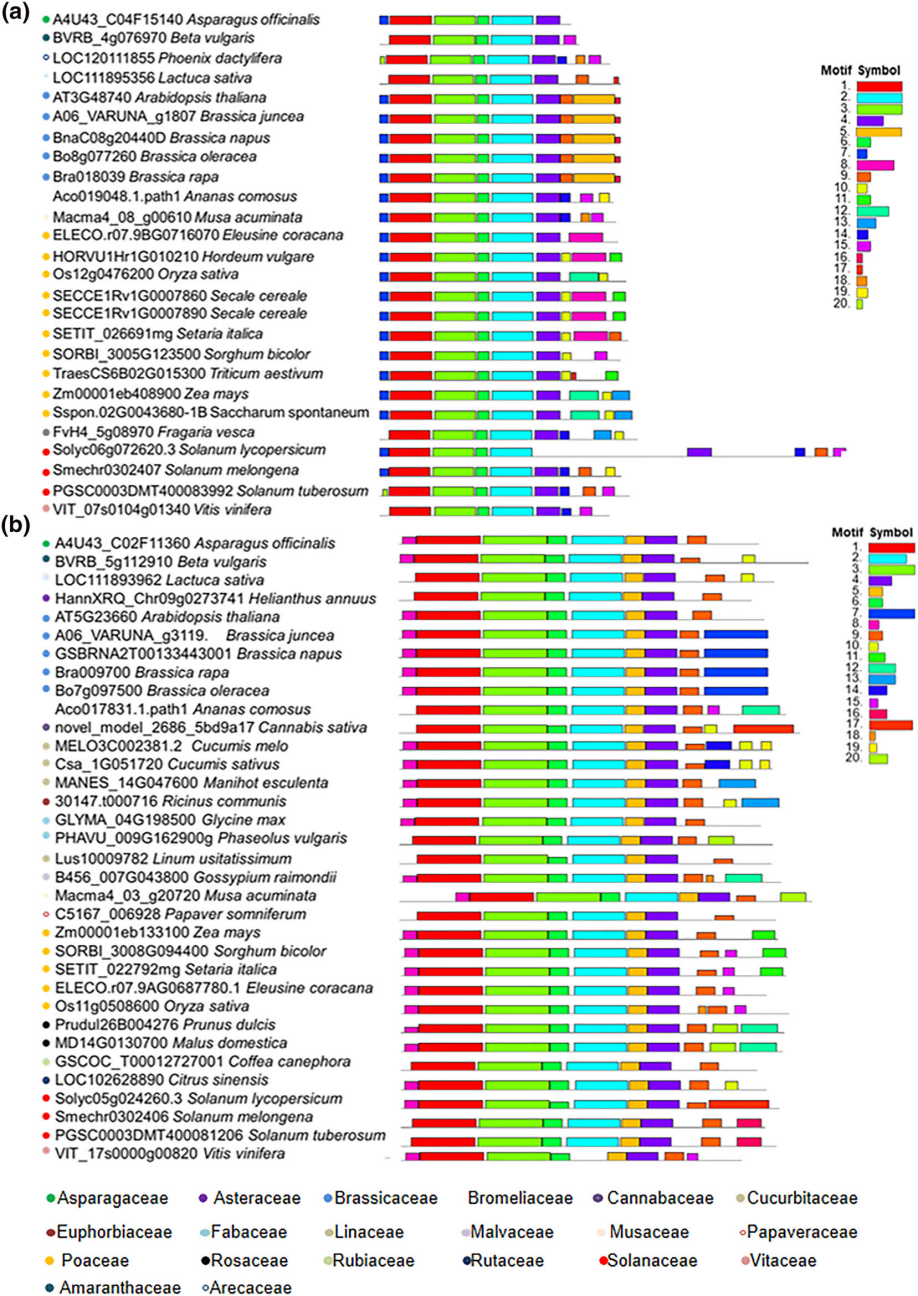
Identification of protein motifs for AtSWEET11 and AtSWEET12 orthologs in different plant species. Protein motifs were identified for the orthologs of (a), AtSWEET11 and (b), AtSWEET12 from 39 different plant species using MEME_suite (https://meme‐suite.org/meme/). The analysis involved 25 protein sequences for (a) and 33 protein sequences for (b). twenty motifs were identified for each, and the motif symbols are indicated by different colors. Families of each species are indicated by colored circles. For more details, see Supplementary File 1.

The length of the protein encoded by *AtSWEET11* and *AtSWEET12* orthologs varies between 209 and 302 amino acids and between 214 and 293 amino acids, respectively (Supplementary File 1b, c). Most AtSWEET11 and AtSWEET12 orthologs carry seven TM helices (TMH) (Supplementary Figure 7 and Supplementary File 1a) except two, namely, *Vitis vinifera* and *S. lycopersicum*, which form six and sixteen TMHs, respectively (Supplementary File 1a). Protein motif analysis revealed the presence of 20 different motifs in both AtSWEET11 and AtSWEET12 orthologs (Figure 5). Of these, motifs 1, 2, 3, and 4 were identified as basic features of the transporter domain of SWEETs, while the other motifs were unannotated. Motifs 1, 2, 3, 4, and 6 were present in AtSWEET11 orthologs from all species. Motifs 1, 3, 4, 5, 6, and 9 were present in AtSWEET12 orthologs from all species (Figure 5 and Supplementary Figure 8). The CTD was highly non‐conserved for AtSWEET11 and AtSWEET12 orthologs from different plant species (Supplementary Figure 9). Recently, it has been shown that phloem parenchyma cell‐specific expression of AtSWEET11 is regulated at the post‐transcriptional level by the two evolutionary duplicated domains in the AtSWEET11 coding sequence along with its promoter including 5′ UTR through RNA‐binding protein (Zhang et al., 2021).

**FIGURE 6:**
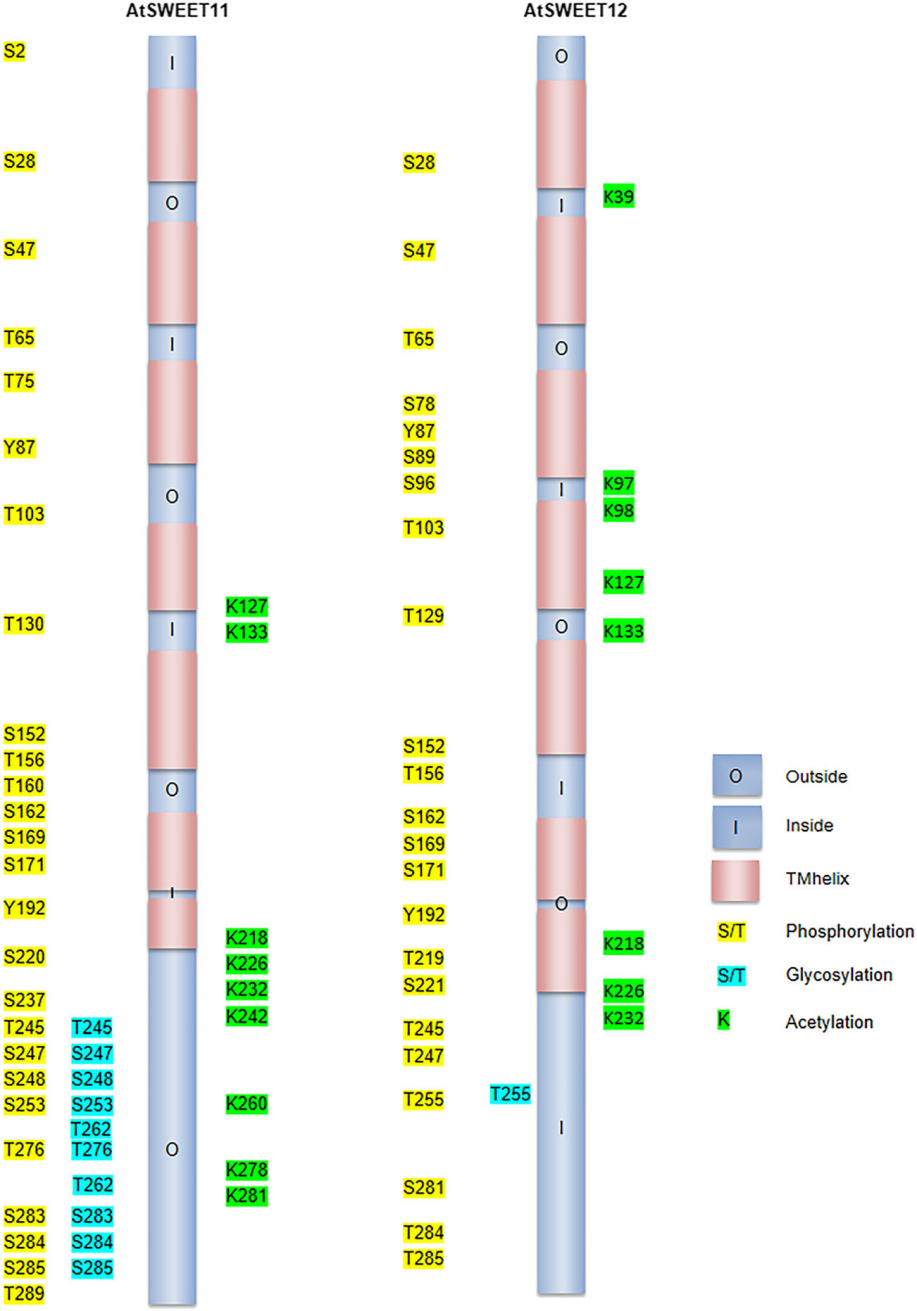
**The prediction of post‐translational modifications of ATSWEET11 and ATSWEET12.** Transmembrane helices in ATSWEET11 and ATSWEET12 are illustrated in peach. PTMs are labeled as follows: In yellow, glycosylation in cyan, and acetylation in green. The phosphorylation, glycosylation and acetylation sites were predicted using NetPhos 3.1, NetOGlyc 4.0 and PAIL server respectively.

Besides, we have identified possible post‐translational modification (PTM) sites for phosphorylation, acetylation, and glycosylation that regulate AtSWEET11 and AtSWEET12 transporters (Figure 6). Membrane phosphoproteome analysis and the presence of a possible phosphorylation site in the CTD of AtSWEET11 (Reiland et al., 2009) strongly indicate phosphorylation‐based regulation. PTM analysis of AtSWEET11 and AtSWEET12 orthologs revealed the presence of potential sites for phosphorylation and acetylation at the CTD in a majority of the analyzed plant species (Supplementary File 1 f, g, h) (Anjali et al., 2020). However, the sites for glycosylation were available for the orthologs of only a few species (Supplementary File 1 f, g, h).

### 3.6 Transcript expression analysis revealed the redundant or exclusive function of *AtSWEET11* and *AtSWEET12* and their orthologs in other plants

**FIGURE 7:**
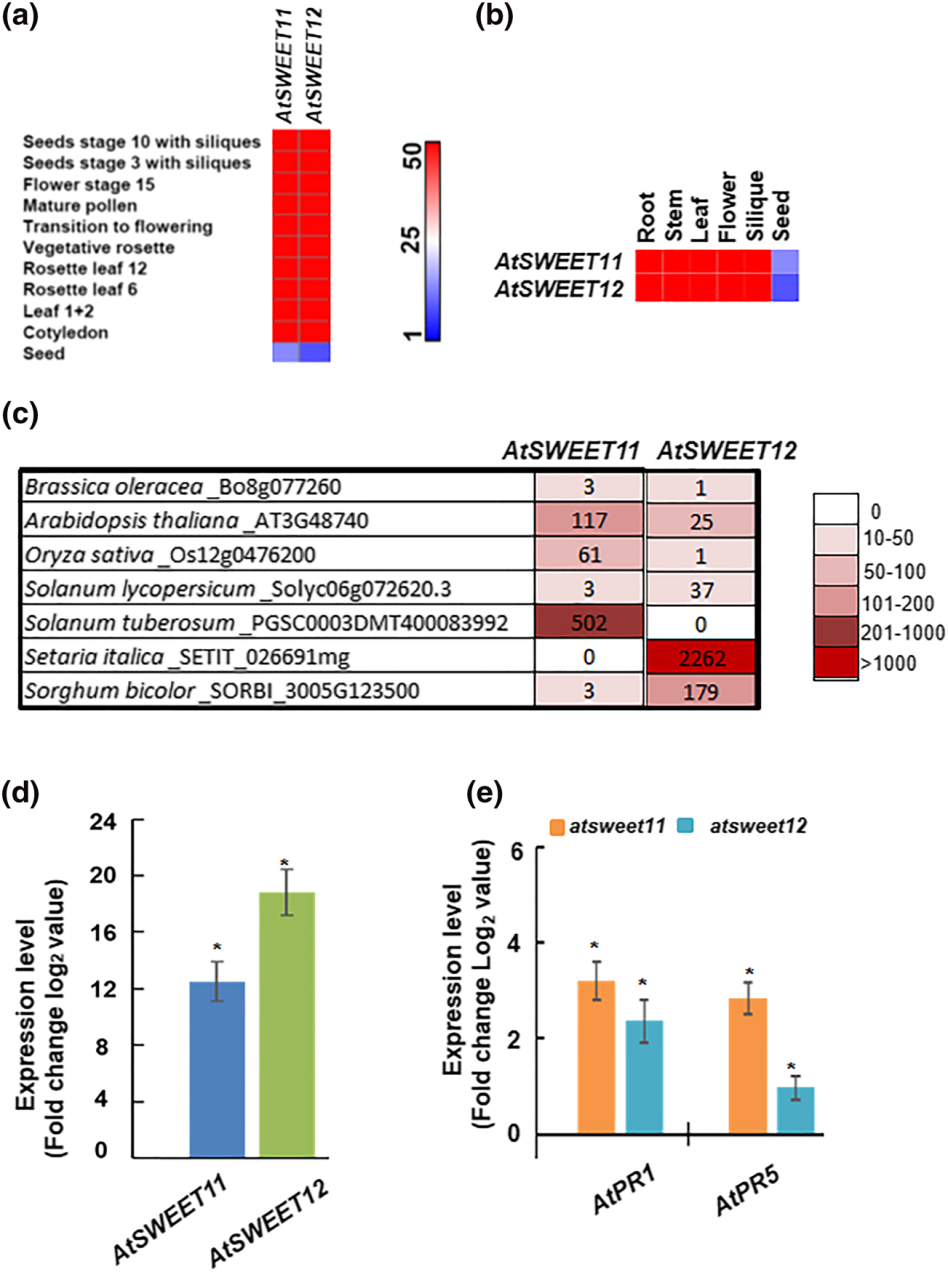
In silico and *in planta* transcript expression analysis of *AtSWEET11 and AtSWEET12* genes*.* (a), the spatial and developmental expression profile of *AtSWEET11 and AtSWEET12* genes during different developmental stages of Arabidopsis. (b), tissue‐specific expression profile of *AtSWEET11 and AtSWEET12* genes in Arabidopsis. (a) and (b), the expression *AtSWEET11 and AtSWEET12* genes were derived from publically available microarray data (http://bbc.botany.utoronto.ca/efp/cgi‐bin/efpWeb.cgi). The absolute values for transcript expression of *AtSWEET11 and AtSWEET12* genes at (a), different stages and (b), different tissues including roots, stems, flowers, siliques and seeds of Arabidopsis wild‐type plants were represented in the form of heat map. Color bar ranging from red to blue indicate the high and low levels of transcript expression respectively. Color bar ranging from red to blue indicate the high and low levels of transcript expression respectively. (c), transcript expression of *AtSWEET11* and *AtSWEET12* orthologs in the leaf tissues from different plant species including *Brassica oleracea* , *Arabidopsis thaliana* , *Oryza sativa* , *Solanum lycopersicum* , *S. tuberosum* , *Triticum aestivum* , *Setaria italica* and *Sorghum bicolor* were obtained from publically available expression atlas (https://www.ebi.ac.uk/gxa/home). The transcript values were expressed as transcripts per millions (TPM) and were represented in the form of heat map. Color bar ranging from dark red to white indicate the levels of transcript expression from high to low or undetected respectively. (d), the transcript expression pattern of *AtSWEET11* and *AtSWEET12* genes after *Pst* DC3000 inoculations is shown here. The 32‐d‐old Arabidopsis wild‐type plants were syringe‐inoculated with sterile water (mock), *Pst* DC3000 at 5 X 10^5^ CFU/ml. samples were collected at 0 and 16 hpi. The transcript levels were measured by RT‐qPCR. For each treatment, the fold change in gene expression levels were calculated over mock‐treated wild‐type samples and were expressed as log2 values. Bars represent the transcript expression pattern of genes. Bars above and below the horizontal axis indicate the upregulation and downregulation in transcript expression respectively. Asterisks indicate significant difference from mock‐treated wild‐type (student's *t* test; *P < .01). Data were obtained from mean of three biological replicates (n = 3) and error bars show ± standard error of mean. (e), the transcript levels of *AtPR1* and *AtPR5* the defense responsive genes in *atsweet11* and *atsweet12* mutants. Leaf samples were collected from 32‐d‐old Arabidopsis wild‐type and mutant plants. The transcript levels were measured by RT‐qPCR. The fold change in expression levels were obtained over wild‐type and expressed as Log_2_ values. Bars represent the transcript expression pattern of *AtPR1* and *AtPR5* genes. Bars above and below the horizontal axis indicate the up‐regulation and down‐regulation in transcript expression respectively. Asterisks (*) indicate a significant difference from wild‐type (student's *t* test; *P < .01). Data were obtained from mean of four biological replicates (n = 4) and error bars show ± standard error of mean.

The in silico spatial and developmental expression profile of *AtSWEET11 and AtSWEET12* genes during different developmental stages of Arabidopsis revealed that these two genes are well expressed at various developmental stages. The expression pattern of these two genes indicates *AtSWEET11 and AtSWEET12* genes are crucial for plant development (Figure 7a). Moreover, the tissue‐specific expression profile of *AtSWEET11 and AtSWEET12* genes in roots, stems, flowers, siliques and seeds of Arabidopsis wild‐type plants were studied and we observed the expression of these two genes in all tissues except seeds (Figure 7b). Taken together, the spatial, developmental and tissue‐specific expression profile suggests that both *AtSWEET11 and AtSWEET12* are involved in various plant developmental and physiological processes and their role could be redundant or exclusive in these processes. Moreover, we have also analyzed the expression of some of the orthologs of *AtSWEET11* and *AtSWEET12* in the leaf tissues of different plants. We found that in the leaves of *O. sativa* and *Solanum tuberosum* the transcript expression of *AtSWEET11* orthologs was high (Figure 7c), while the expression of *AtSWEET12* orthologs remained undetected (Figure 7c). While, in *Setaria italica* and *Sorghum bicolor*, the expression of *AtSWEET12* orthologs was high (Figure 7c) and *AtSWEET11* orthologs remained undetected (Figure 7c). The differential expression pattern of *AtSWEET11* and *AtSWEET12* orthologs indicate distinct and exclusive roles of these transporters in different plant species.

Further, we have studied the involvement of *AtSWEET11* and *AtSWEET12* genes in plant‐pathogen interactions by performing the transcript expression analysis of these genes in Arabidopsis after inoculation with *P. syringae* pv. *tomato* DC3000 (*Pst* DC3000). We observed the increase in the transcript levels of the *AtSWEET11* and *AtSWEET12* genes in wild‐type plants after *Pst* DC3000 inoculation compared to mock (Figure 7d). Besides, we have checked the transcript levels of the defense responsive genes including *pathogenesis‐related (PR)* genes, *AtPR1* and *AtPR5* in *atsweet11* and *atsweet12* mutant plants. We observed the increase in the transcript levels of *AtPR1* and *AtPR5* in *atsweet11* and *atsweet12* mutant plants compared to wild‐type plants (Figure 7e). These results indicate the involvement of both *AtSWEET11* and *AtSWEET12* genes during biotic stress especially after infection with foliar bacterial pathogens.

## 4 DISCUSSION

**FIGURE 8:**
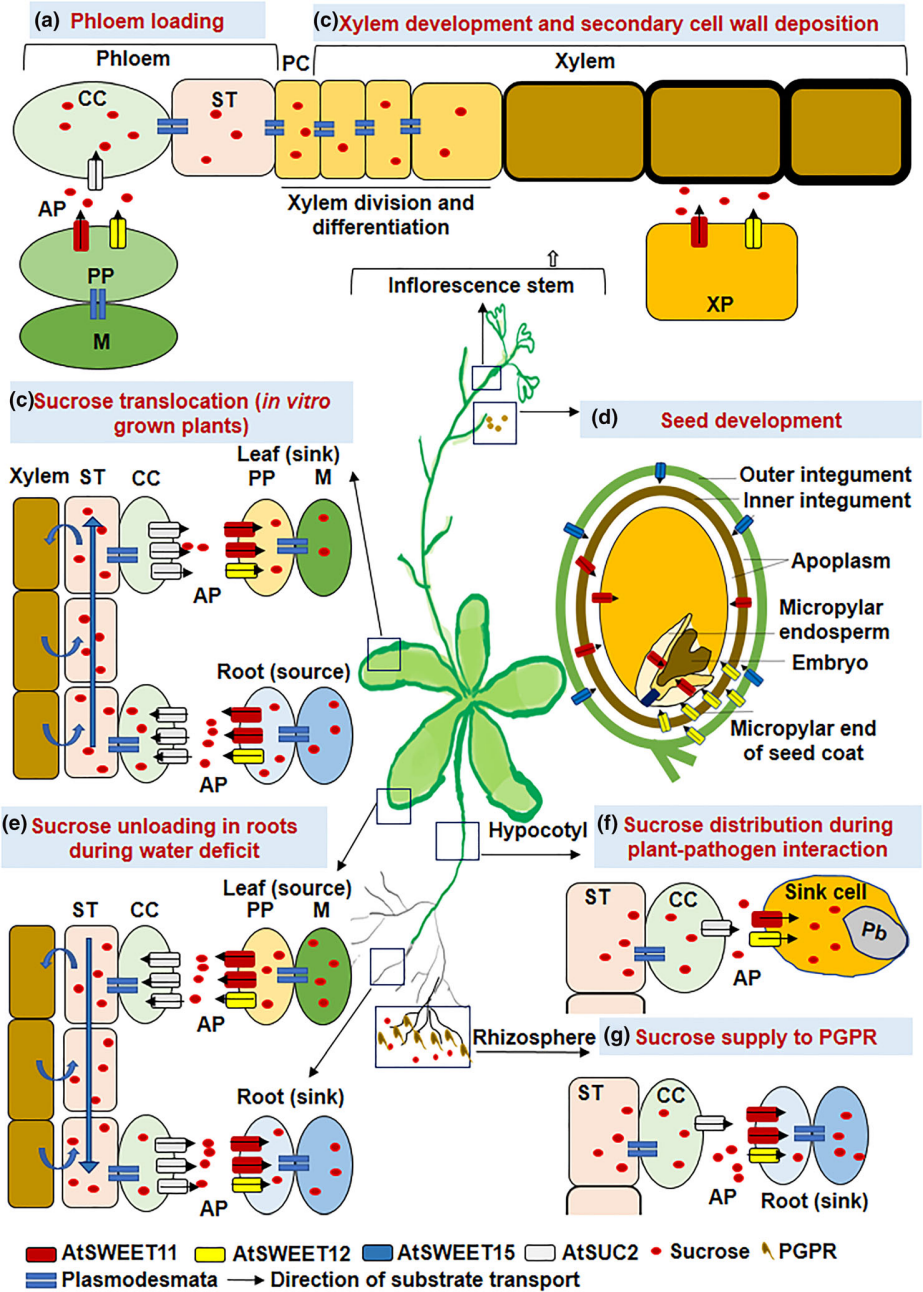
AtSWEET11 and AtSWEET12 localization, involvement in the sucrose transport pathway, and related physiological functions and stress responses in Arabidopsis. AtSWEET11 and AtSWEET12 transporters are reported to play a major role in the roots, hypocotyl, leaf/stem (tracheid), and seeds. (a), during apoplasmic phloem loading, phloem parenchyma‐localized AtSWEET11 and AtSWEET12 transport sucrose from the mesophyll cells to the phloem apoplast. next, AtSUC2 transports the sucrose from the apoplast to the sieve element–companion cell complex (Chen et al., 2012). (b), in in vitro grown plants with exogenous carbon supply, the roots act as a source tissue, and the leaves become the sink. The directionality of sucrose transport by AtSWEET11 and AtSWEET12 gets reversed according to the sucrose concentration gradient, i.e., from the root (source) to the leaf tissues (sink) (Papaioannou, 2018). (c), during xylem development, AtSWEET11 and AtSWEET12 are localized in xylem parenchyma cells in the inflorescence stem and facilitate secondary cell wall formation by delivering carbon sources to the developing xylem cells in the inflorescence (Le Hir et al., 2015). (d), during seed filling, AtSWEET15 localized in the outer integument transports sucrose into the apoplast. AtSWEET11 localized in the inner integument facilitates sucrose supply to the developing embryo from the micropylar endosperm. AtSWEET12 localized in the micropylar end of the seed coat facilitates sucrose transport to the seed coat region. Together, these three transporters, among others, are involved in sink‐drawing ability during seed development/grain filling (Chen et al., 2015). (e), during water deficit conditions, AtSWEET11, AtSWEET12, and AtSUC2 are involved in drawing more sucrose to the root cells. Under water stress, these transporters accumulate in the roots and unload sucrose from the apoplast to the sink cells in the roots and facilitate root growth by allocating more sucrose from the leaves to the roots (Durand et al., 2016). (f), AtSWEET11 and AtSWEET12 facilitate sugar delivery towards the pathogen (here, *Plasmodiophora brassicae* at the site of infection in the hypocotyl region) (Walerowski et al., 2018). (g), AtSWEET11 and AtSWEET12 participate in phloem unloading of sucrose, especially to the lateral roots. These transporters control the sugar supply from the shoot to the root and then distribute sugars to plant growth‐promoting rhizobacteria (PGPR, i.e., here, *Pseudomonas simiae* WCS417r in the rhizosphere region) (Desrut et al., 2020). CC: Companion cell; ST: Sieve tubes; PP: Phloem parenchyma; M: Mesophyll cells; PC: Pro‐cambium cells; XP: Xylem parenchyma; AP: Apoplast; Pb; *P. brassicae.*

The AtSWEET11 and AtSWEET12 are crucial transporters among the entire Arabidopsis gene family members. Initially the role of these transporters was identified in apoplasmic phloem loading. AtSWEET11 and AtSWEET12 proteins are localized on the plasma membrane of the phloem parenchyma cells in leaf tissue, where they are involved in the translocation of sucrose from mesophyll cells to the phloem apoplasm (Chen et al., 2012). The sucrose effluxed into the phloem apoplasm is transported to the sieve element–companion cell (SE/CC) complex with the help of the sucrose transporter AtSUC2 against a concentration gradient (Ayre, 2011; Giaquinta, 1983; Srivastava et al., 2008; Srivastava, Dasgupta, et al., 2009; Srivastava, Ganesan, et al., 2009). Chen et al. (2012) showed that plants carrying mutations in both *AtSWEET11* and *AtSWEET12* showed moderate growth defects and accumulated excessive sugar in the leaves due to blockage in the sugar translocation pathway. However, single mutants of either of these genes did not affect the plant (Chen et al., 2012), implying a redundant function of AtSWEET11 and AtSWEET12 in sucrose transport during phloem‐loading process (Figure 8a). AtSWEET11 and AtSWEET12 are also involved in long‐distance translocation of sucrose in both the source and sink of plants grown in vitro (Figure 8b) (Papaioannou, 2018). The study suggests that the directionality of sucrose transport by AtSWEET11 and AtSWEET12 can be reversed in accordance to the sucrose concentration gradient. Besides, the study showed that the transgenic with the single mutant *atsweet12* and *AtSWEET11* overexpression translocated more sucrose from the source (root tissues) to the sink (leaf tissues). However, this was not observed in the reverse case, i.e., *atsweet11* mutant with *AtSWEET12* overexpression (Papaioannou, 2018). In other words, in the absence of AtSWEET12, sucrose transport is overtaken by AtSWEET11 but not vice versa; hence, AtSWEET11 is crucial for sucrose transport. Similarly, in another study, endogenously supplied sucrose—by providing high light conditions—showed contrasting effects on the expression of *AtSWEET11* and *AtSWEET12* (Wei et al., 2020). Thus, in the presence of high endogenous sucrose levels in leaf tissues, *AtSWEET11* expression is upregulated, but *AtSWEET12* expression is not. Taken together, these studies argue against the redundant roles of AtSWEET11 and AtSWEET12 under certain conditions and hint at the dominant role of AtSWEET11 in sucrose transport function. Our molecular docking and molecular dynamics (MD) simulation study revealed the central cavity and the middle of the TM region as a sucrose binding site for both AtSWEET11 and AtSWEET12 (Supplementary Figure 3) (Figure 1c and f), which is similar to the already known crystal structure of AtSWEET13‐dCMP complex. Interestingly, both AtSWEET11 and AtSWEET12 transporters share the similar residues which interact with sucrose and also equivalent residues interacting with dCMP in the reported AtSWEET13 structure (Figure 1i and j, Supplementary Table 1). This is to allow the alternate access to the sucrose molecule from either side of the membrane during transport.

Besides their phloem‐loading functions, AtSWEET11 and AtSWEET12 facilitate secondary cell wall formation by delivering carbon sources to developing xylem cells (Le Hir et al., 2015) (Figure 8c). Phenotypic and anatomical characteristics revealed reduced stem diameter and xylem and phloem areas in single *atsweet11* and double *atsweet11;12* mutants, but no distinguishable features were observed in *atsweet12* mutant. These observations established that the mutation of *AtSWEET11* has a dominant effect on the phenotype compared to the mutation of *AtSWEET12* alone. However, the double mutant phenotype is more prominent than that of single mutants (Le Hir et al., 2015), suggesting that AtSWEET11 and AtSWEET12 transporters work in tandem to regulate sugar transport. Moreover, AtSWEET11, AtSWEET12, and AtSWEET15 are crucial for seed filling in Arabidopsis (Chen et al., 2015) (Figure 8d). AtSWEET11 and AtSWEET12 also regulates the sugar flux in response to fluctuating environments. The transcript levels of the *AtSWEET11*, *AtSWEET12*, and *AtSUC2* genes are highly induced in the leaves of plants under water deficit conditions and these sucrose transporters were predicted to be involved in exporting sucrose accumulated in the leaves to the roots (Durand et al., 2016). In the recent detailed study, the phosphorylation of these sucrose transporters was shown to direct enhanced root:shoot ratio in plants under drought stress (Chen et al., 2022; Gong & Yang, 2022) (Figure 8e). AtSWEET11 and AtSWEET12 however, appear to be the most important, because they play a crucial role in all these essential developmental and physiological processes. In the present study, we detailed the information on the orthologs of *AtSWEET11* and *AtSWEET12* transporters in 39 economically important plants. A few of the species, out of 39 were having orthologs for either *AtSWEET11* or *AtSWEET12* indicating that during the course of evolution, only one transporter might have been responsible for the sugar transport function.

Besides, the length of protein encoded by *AtSWEET11* and *AtSWEET12* orthologs varies between 209 and 302 amino acids and between 214 and 293 amino acids, which seems to be comparable to that reported for AtSWEET11 and AtSWEET12 in Arabidopsis. Most AtSWEET11 and AtSWEET12 orthologs carry seven TM helices (TMH) and 20 different protein motifs comparable to that reported for AtSWEET11 and AtSWEET12. The in silico spatial, developmental and tissue‐specific expression profile of Arabidopsis *AtSWEET11 and AtSWEET12* genes revealed that these two genes are well expressed in all tissue and required during various plant developmental and physiological processes. However, the expression level for the orthologs of *AtSWEET11* and *AtSWEET12* was varying in the leaf tissues of different plants. The transcript expression of *AtSWEET11* orthologs was high and *AtSWEET12* orthologs remained undetected in *O. sativa* and *S. tuberosum* and vice‐versa for *S. italica* and *S. bicolor* (Figure 7c). The *AtSWEET11* and *AtSWEET12* orthologs might have distinct and exclusive roles in these plant species rather than redundant function.

Moreover, studies have indicated the involvement of AtSWEET11 and AtSWEET12 transporters in plant‐pathogen interactions. Chen et al. (2010) reported the induced transcript levels of *AtSWEET11* and *AtSWEET12* genes during infection with biotrophic, hemibiotrophic, and necrotrophic pathogens. Here, we report the increase in the transcript levels of the *AtSWEET11* and *AtSWEET12* genes after *Pst* DC3000 inoculation and increase in the transcript levels of the defense responsive genes *AtPR1* and *AtPR5* in *atsweet11* and *atsweet12* mutant plants suggesting the involvement of both AtSWEET11 and AtSWEET12 during biotic stress especially after infection with foliar bacterial pathogens (Figure 7d and e). Gebauer et al. (2017) showed the involvement of AtSWEET11 and AtSWEET12 transporters after infection with the fungal hemibiotroph *Colletotrichum higginsianum*. Their study showed that *atsweet11;12* double mutants, but not the single mutants, were resistant to *C. higginsianum* due to sugar‐mediated priming of the salicylic acid pathway. However, the strong accumulation of AtSWEET12‐YFP fusion proteins at the localized infection sites, but not of AtSWEET11‐YFP fusion proteins, does not support the redundant roles of AtSWEET11 and AtSWEET12 transporters. The Arabidopsis interaction with the protist biotroph *Plasmodiophora brassicae* established the role of AtSWEET11 and AtSWEET12 transporters in facilitating sugar delivery towards pathogens at the infection site (Walerowski et al., 2018) (Figure 8f). Transcript expression of *AtSWEET11* and *AtSWEET12* was highly induced after inoculation of Arabidopsis with plant growth‐promoting rhizobacteria (PGPR) strain *Pseudomonas simiae* WCS417r, and the beneficial effect of *P. simiae* was lost in Arabidopsis *atsweet11;12* double mutants (Desrut et al., 2020). This suggests that AtSWEET11 and AtSWEET12 could possibly function in controlling the sugar supply from the shoot to the root and its distribution to the PGPR, which might positively impact the plant–PGPR interaction (Figure 8g). Similarly, in other study AtSWEET12 was shown to suppress multiplication of different species of *P. syringae* by restricting sucrose availability to these foliar bacterial pathogens in the leaf apoplast. The study traced the AtSWEET11‐mediated sucrose flux to be modulated through AtSWEET12 via plasma membrane targeting and an oligomerization‐dependent regulatory mechanism in Arabidopsis. This also indicates the exclusive role of AtSWEET12 in suppressing bacterial multiplication and the role of AtSWEET11 in supplying sugars to bacterial pathogens in the apoplast (Fatima & Senthil‐Kumar, 2021). In the present study, a large number of cis‐acting elements related to defense and biotic stress, such as MYB and MYC elements were abundantly distributed in the promoter sequences of *AtSWEET11* and *AtSWEET12* orthologs from different plant species, followed by STRE, ARE, LTR, W box, WUN, and MBS elements (Figure 4). Nevertheless, future studies are required to validate the role of *AtSWEET11* and *AtSWEET12* orthologs from other plants during plant–pathogen interactions.

## 5 CONCLUSION AND FUTURE DIRECTIONS

AtSWEET11 and AtSWEET12 transporters function in tandem to modulate sugar flux in Arabidopsis. The present study explored the homology models of AtSWEET11 and AtSWEET12 and revealed the key amino acids essential for substrate recognition and transport. Docking studies showed that the central sucrose binding residues are almost similar in both AtSWEET11 and AtSWEET12 transporters. We deduced that the structural similarities between AtSWEET11 and AtSWEET12 is responsible for the functional redundancy of these two transporters. However, it is interesting to note that the CTD varies between AtSWEET11 and AtSWEET12 and their orthologs in different plant species. It is highly plausible that these transporters are independently regulated through PTM at the CTD, which might be a reason for distinct and exclusive roles of these two transporters in various plant physiological processes. The differential expression of *AtSWEET11* and *AtSWEET12* orthologs also support their specific roles in different plant species. Further in‐vitro assays and *in planta* studies are required to shed light on the redundant or exclusive functions of the *AtSWEET11* and *AtSWEET12* orthologs from different plant species. More studies are needed to understand the regulation of these two transporters in regulating the sugar levels in response to pathogen infection. Although research on SWEETs has increased considerably, the mechanism of regulation of *AtSWEET11* and *AtSWEET12* orthologs in crop plants during various developmental stages and abiotic and biotic stress conditions remains to be elucidated in greater detail. The structure–function relationship of AtSWEET11 and AtSWEET12 and their orthologs deciphered from the experimentally determined structures with their corresponding native sugar substrates would help gain deeper insights into the mechanistic details of the transport. Finer details on the orthologs will help implement experimental concepts from model plant to crop plants to develop improved crop phenotypes with enhanced productivity and resilience.

## CONFLICT OF INTEREST

The authors declare that they have no known competing financial interests or personal relationships that could have appeared to influence the work reported in this paper.

## AUTHOR CONTRIBUTION STATEMENT

MS‐K conceived the concept and provided outline. UF contributed to the figures, supplemental information and drafted the entire manuscript. DB, WAK and AA contributed to the structure analysis and related part of the manuscript. MK contributed to motif analysis, cis element analysis and related supplementary figures. JV involved in editing the manuscript. MS‐K edited and finalized the manuscript.

## Supporting information


**Supplementary Figure 1.** The Molecular dynamics (MD) trajectory analysis for AtSWEET11 and AtSWEET12
Supplementary Figure 2. Structural superposition of AtSWEET11 and AtSWEET12.
Supplementary Figure 3. RMSD plot of AtSWEET11 and AtSWEET12 for protein backbone and sucrose as a ligand.
Supplementary Figure 4. Multiple sequence alignment of AtSWEET11, AtSWEET12, AtSWEET13, and OsSWEET2b.
Supplementary Figure 5. The time‐tree analysis for evaluating the divergence time of different species used in this study.
Supplementary Figure 6. Tertiary structure prediction of AtSWEET11 and AtSWEE12 orthologs from different plant species.
Supplementary Figure 7. Details of logos of each protein motif for AtSWEET11 and AtSWEET12 orthologs in different plant species.
Supplementary Figure 8. The C‐terminal analysis of AtSWEET11 and AtSWEET12 protein orthologs from different plant species.
Supplementary Figure 9. Flowchart for identifying the orthologous genes for AtSWEET11 and AtSWEET12.

**Supplementary Table 1:** Sucrose‐interacting residues in AtSWEET11 and AtSWEET12 with equivalent dCMP‐binding residues in the AtSWEET13 crystal structure.

**Supplementary Table 2:** Details of conserved residues among AtSWEETs and OsSWEET2b.

**Supplementary File S1.** Details regarding raw data used for figure preparation.
