# Supplementary material for: AtSWEET11 and AtSWEET12 transporters function in tandem to modulate sugar flux in plants
Source: Plant Direct. 2023 Mar 8;7(3):e481. doi: 10.1002/pld3.481 (PMC9995347; doi:10.1002/pld3.481)
Supplement: Supplementary file 1 — Supplementary Figure 1. The Molecular dynamics (MD) trajectory analysis for AtSWEET11 and AtSWEET12 Supplementary Figure 2. Structural superposition of AtSWEET11 and AtSWEET12. Supplementary Figure 3. RMSD plot of AtSWEET11 and AtSWEET12 for protein backbone and sucrose as a ligand. Supplementary Figure 4. Multiple sequence alignment of AtSWEET11, AtSWEET12, AtSWEET13, and OsSWEET2b. Supplementary Figure 5. The time‐tree analysis for evaluating the divergence time of different species used in this study. Supplementary Figure 6. Tertiary structure prediction of AtSWEET11 and AtSWEE12 orthologs from different plant species. Supplementary Figure 7. Details of logos of each protein motif for AtSWEET11 and AtSWEET12 orthologs in different plant species. Supplementary Figure 8. The C‐terminal analysis of AtSWEET11 and AtSWEET12 protein orthologs from different plant species. Supplementary Figure 9. Flowchart for identifying the orthologous genes for AtSWEET11 and AtSWEET12. [file PLD3-7-e481-s002.pdf]

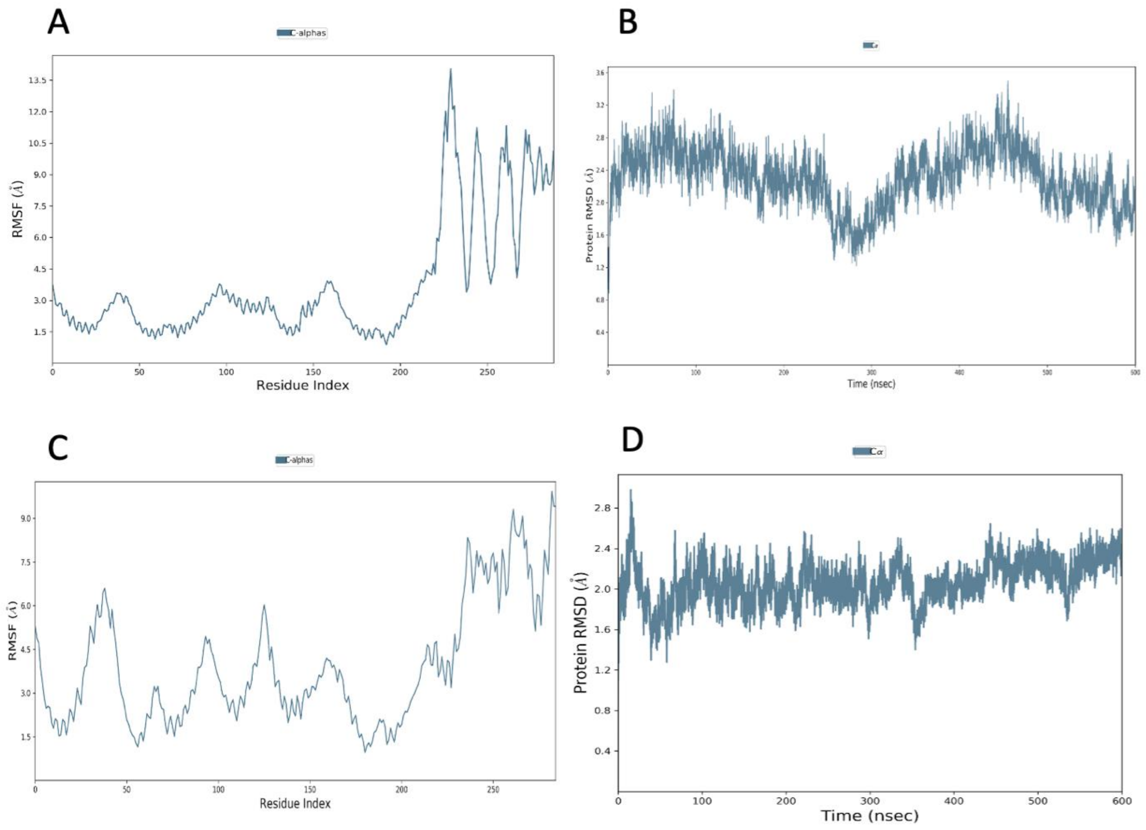


**Supplementary Figure 1. The Molecular dynamics (MD) trajectory analysis for AtSWEET11 and AtSWEET12 A,** RMSF plot for full length AtSWEET11. **B,** RMSD plot for the TM region of AtSWEET11 **C,** RMSF plot for full length AtSWEET12 **D,** RMSD plot for the TM region of AtSWEET12.

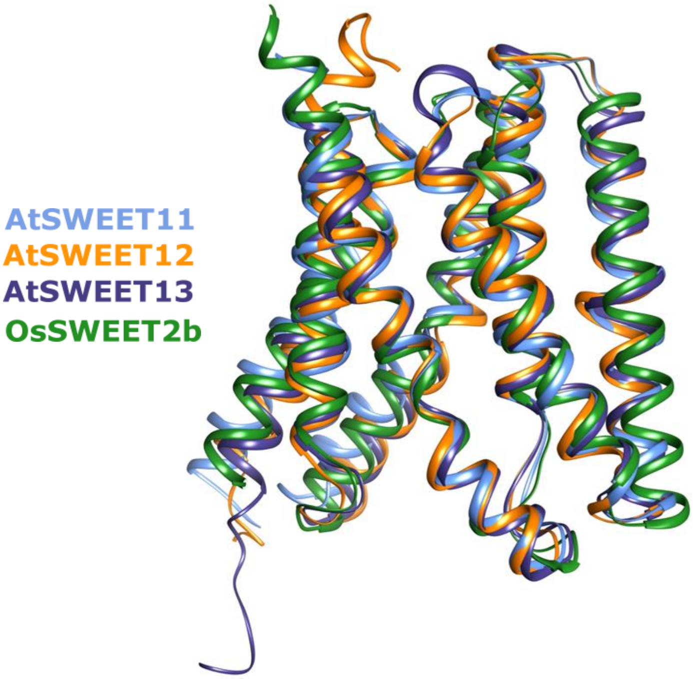


**Supplementary Figure 2. Structural superposition of AtSWEET11, AtSWEET12, AtSWEET13 and OsSWEET2b.** Superposition of homology models of AtSWEET11 (cornflower blue) and AtSWEET12 (orange) with crystal structures of AtSWEET13 (violet) and OsSWEET2b (forest green). The RMSD values of superposition of AtSWEET11 with AtSWEET13 and OsSWEET2b is 1.66 Å and 2.56 Å respectively. The RMSD values of superposition of AtSWEET12 with AtSWEET13 and OsSWEET2b is 1.36 Å and 2.50 Å respectively. For structural superposition, AtSWEET13 (PDB: 5XPD Chain A 1-222 residues), OsSWEET2b (PDB: 5CTG Chain A 1-215 residues) and homology models of AtSWEET11 and AtSWEET12 (1-219 residues) were used. RMSD values were calculated through SSM superposition in COOT. For more details see Supplementary Figure 1

A B

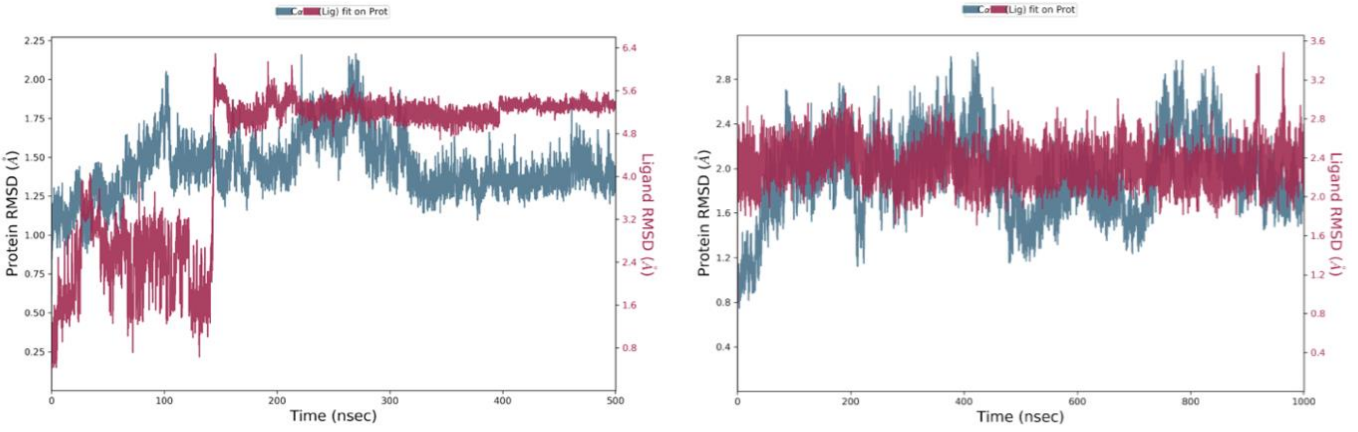


**Supplementary Figure 3. The RMSD plot of AtSWEET11 and AtSWEET12 for protein backbone and sucrose as ligand. A,** The RMSD plot of AtSWEET11. **B,** The RMSD plot of AtSWEET12.

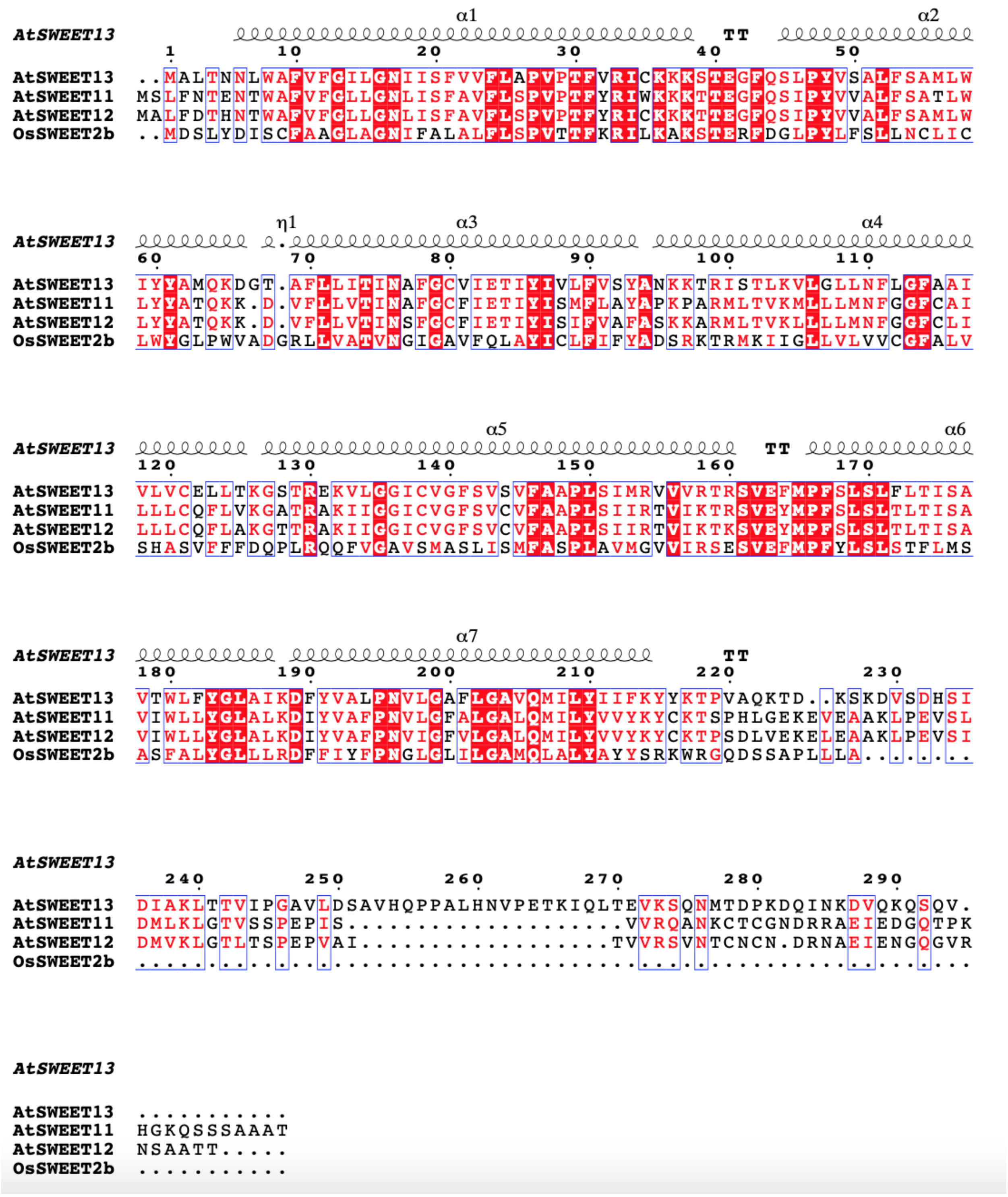

**Supplementary Figure 4. Multiple sequence alignment of AtSWEET11, AtSWEET12, AtSWEET13 and OsSWEET2b.**

The full-length amino acid sequences of AtSWEET11, AtSWEET12, AtSWEET13 and OsSWEET2b were obtained from TAIR (https://www.arabidopsis.org) Sequences were aligned using ClustalW (https://www.genome.jp/tools-bin/clustalw). Secondary structure assignments of AtSWEET13 are indicated above the alignment. Multiple sequence alignment of AtSWEET11, AtSWEET12 with AtSWEET13 and OsSWEET2b showed a total of 61 conserved residues. The conserved residues are highlighted in red.

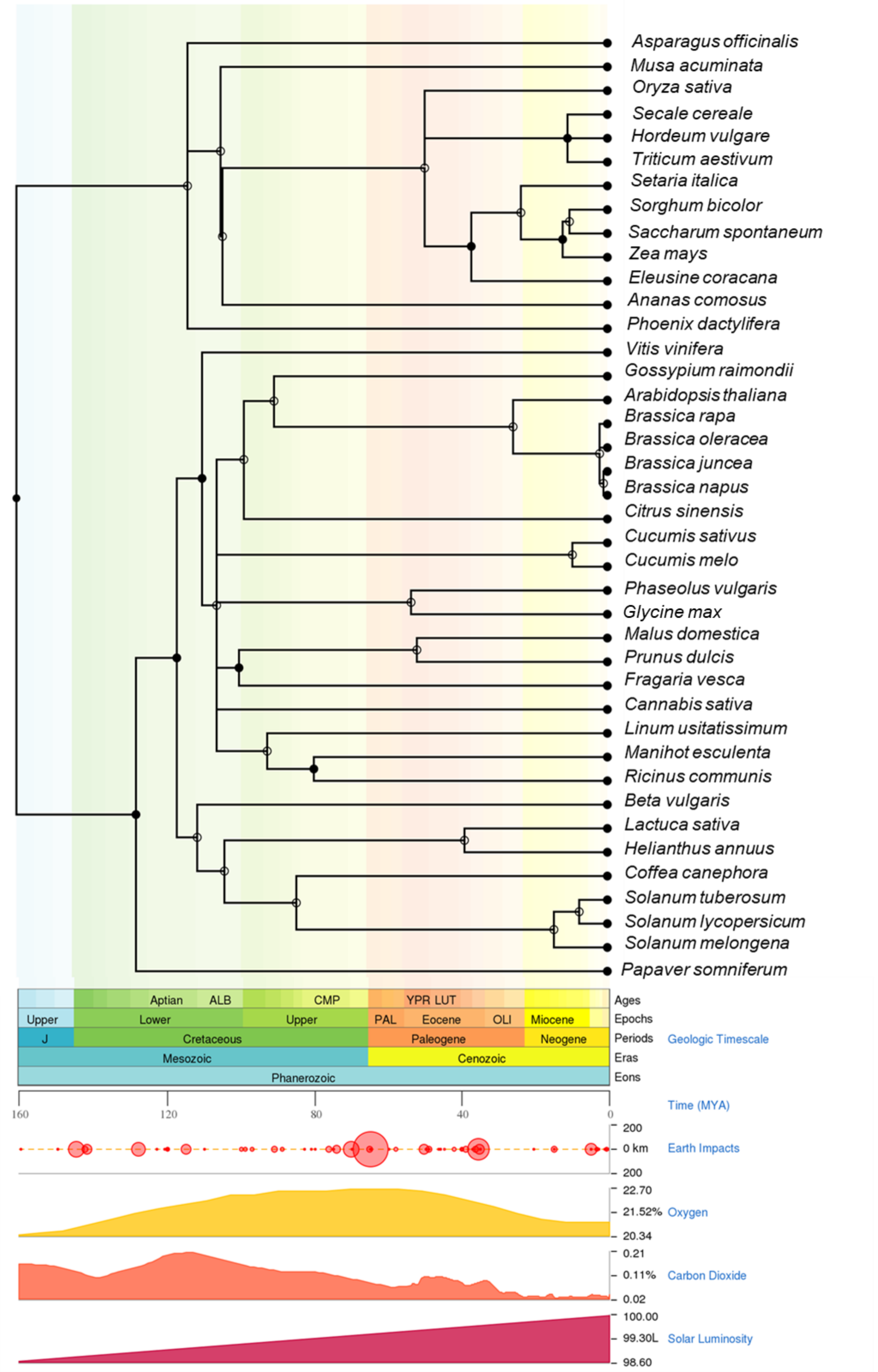
Asparagus officinalis
Musa acuminata
Oryza sativa
Secale cereale
Hordeum vulgare
Triticum aestivum
Setaria italica
Sorghum bicolor
Saccharum spontaneum
Zea mays
Eleusine coracana
Ananas comosus
Phoenix dactylifera
Vitis vinifera
Gossypium raimondii
Arabidopsis thaliana
Brassica rapa
Brassica oleracea
Brassica juncea
Brassica napus
Citrus sinensis
Cucumis sativus
Cucumis melo
Phaseolus vulgaris
Glycine max
Malus domestica
Prunus dulcis
Fragaria vesca
Cannabis sativa
Linum usitatissimum
Manihot esculenta
Ricinus communis
Beta vulgaris
Lactuca sativa
Helianthus annuus
Coffea canephora
Solanum tuberosum
Solanum lycopersicum
Solanum melongena
Papaver somniferum
Aptian ALB CMP YPR LUT
Ages
Upper Lower Upper PAL Eocene OLI Miocene
Epochs
J Cretaceous Paleogene Neogene
Periods
Geologic Timescale
Mesozoic Cenozoic
Eras
Phanerozoic
Eons
160 120 80 40 0
Time (MYA)
200
0 km
200
Earth Impacts
22.70
21.52%
20.34
Oxygen
0.21
0.11%
0.02
Carbon Dioxide
100.00
99.30L
98.60
Solar Luminosity

**Supplementary Figure 5. The time-tree analysis for evaluating the divergence time of different species used in this study.**

The phylogenetic analysis of thirty-nine different plant species from twenty different families was performed. The species names were taken as input and  time-tree was generated using the MEGA X (1). The nodes in the tree indicate the divergence times for different plant species. The complete list of the species can obtained from Supplementary File 1.

Kumar S., Stecher G., Li M., Knyaz C., and Tamura K. (**2018**). MEGA X: Molecular Evolutionary Genetics Analysis across computing platforms. *Molecular Biology and Evolution* **35**:1547-1549.

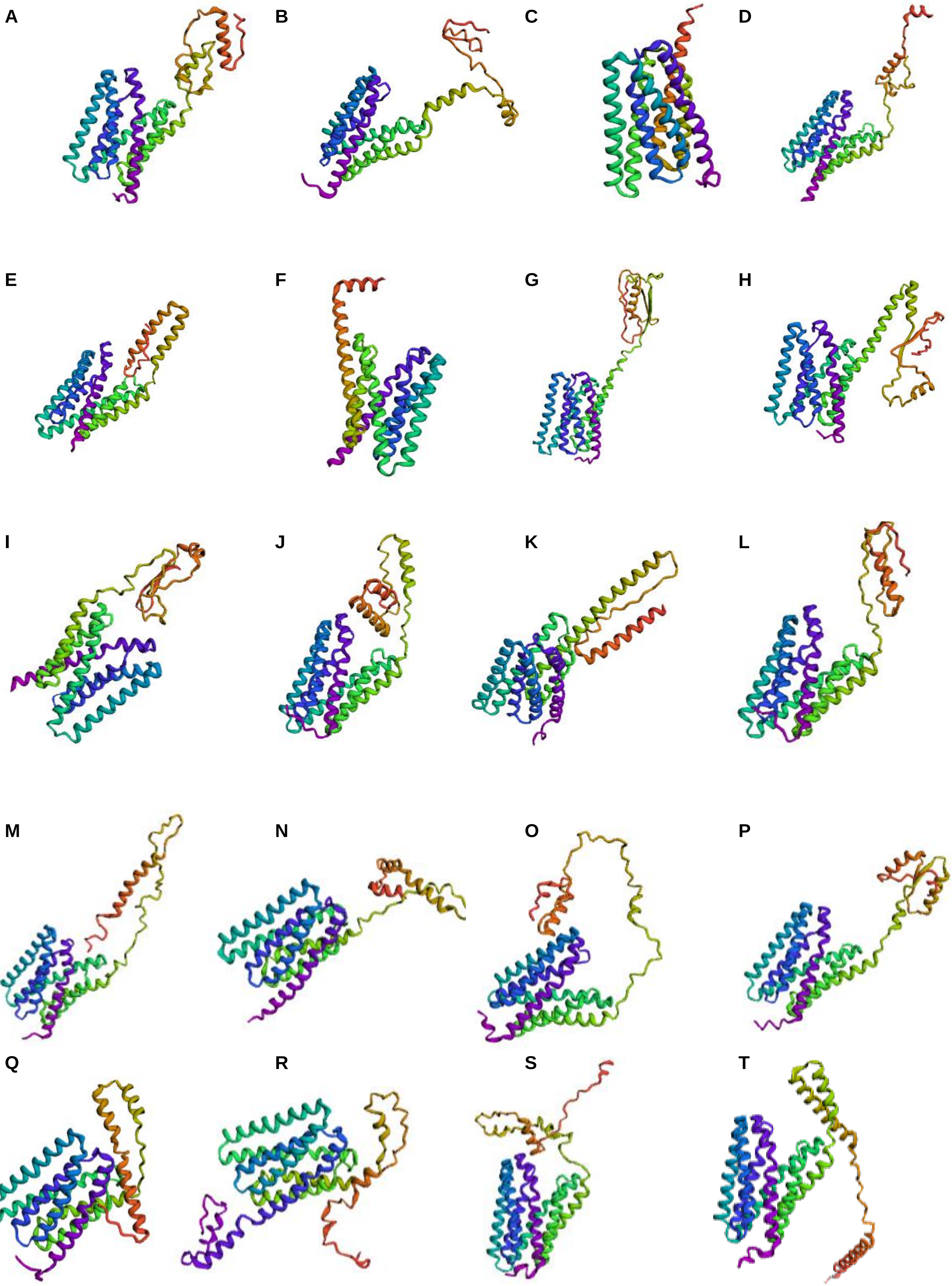
A
B
C
D
E
F
G
H
I
J
K
L
M
N
O
P
Q
R
S
T

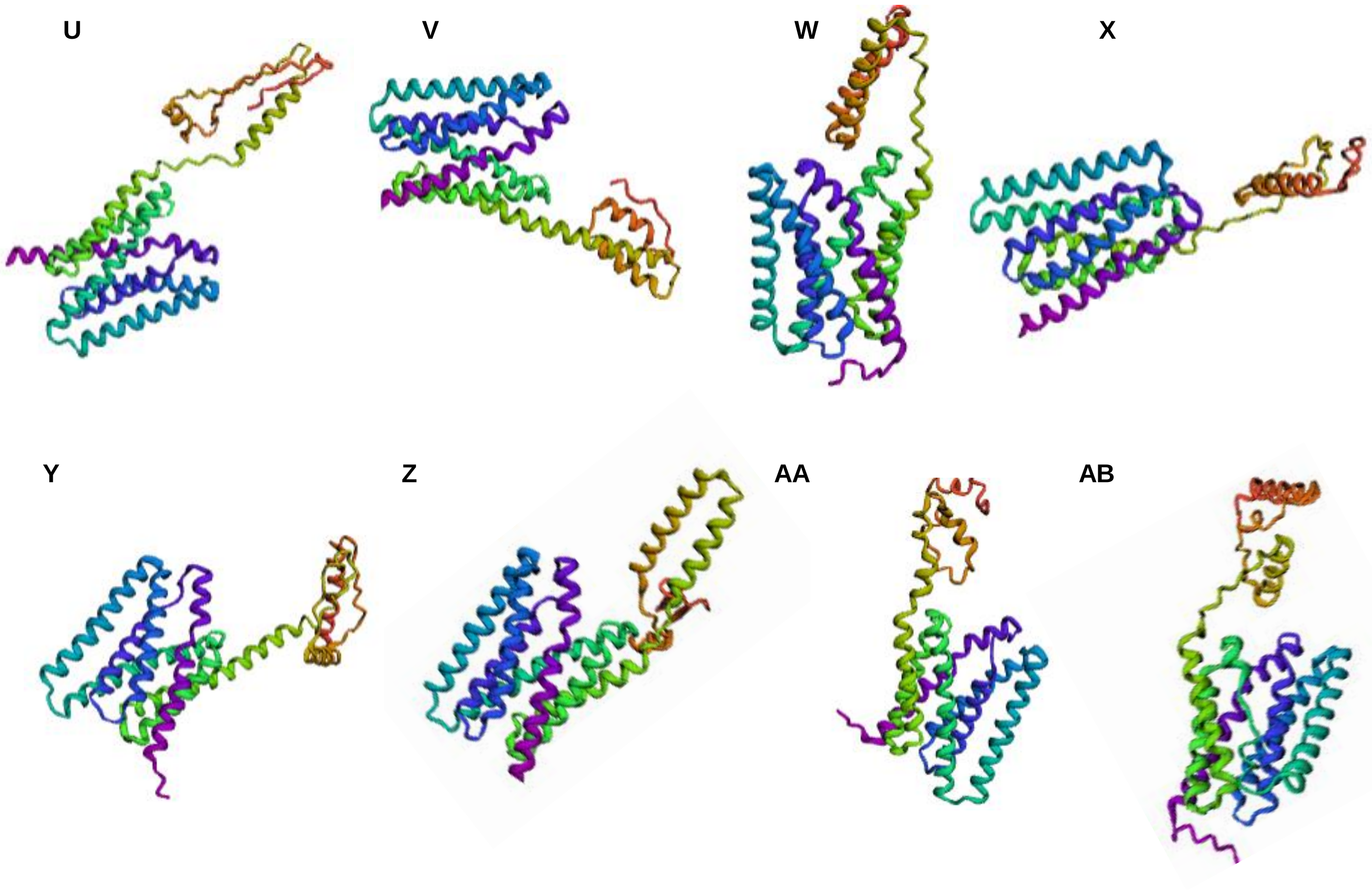


**Supplementary Figure 6. Tertiary structure prediction of AtSWEET11 and AtSWEE12 orthologs from different plants.** The tertiary structures of the proteins orthologus to SWEET11 and SWEET12 were predicted using Robetta (https://robetta.bakerlab.org/). The structures depicted in the figure represents a family A,B are from Brassicaceae C,D Asparagaceae E Asteraceae F,G Amaranthaceae H Cannabaceae I,J Bromeliaceae K Cucurbitaceae L Euphorbiaceae M Fabaceae N Linaceae

O Malvaceae P Papaveraceae Q,R Musaceae S,T Poaceae U Rosaceae V Rubiaceae W Rutaceae X Arecaceae Y,Z Solanaceae and AA, AB Vitaceae

This tertiary structure prediction analysis revealed that the majority of SWEET protein C-terminal regions are intrinsically disordered and the C-terminal region did not exhibits any ordered structure.

**A** **SWEET11**

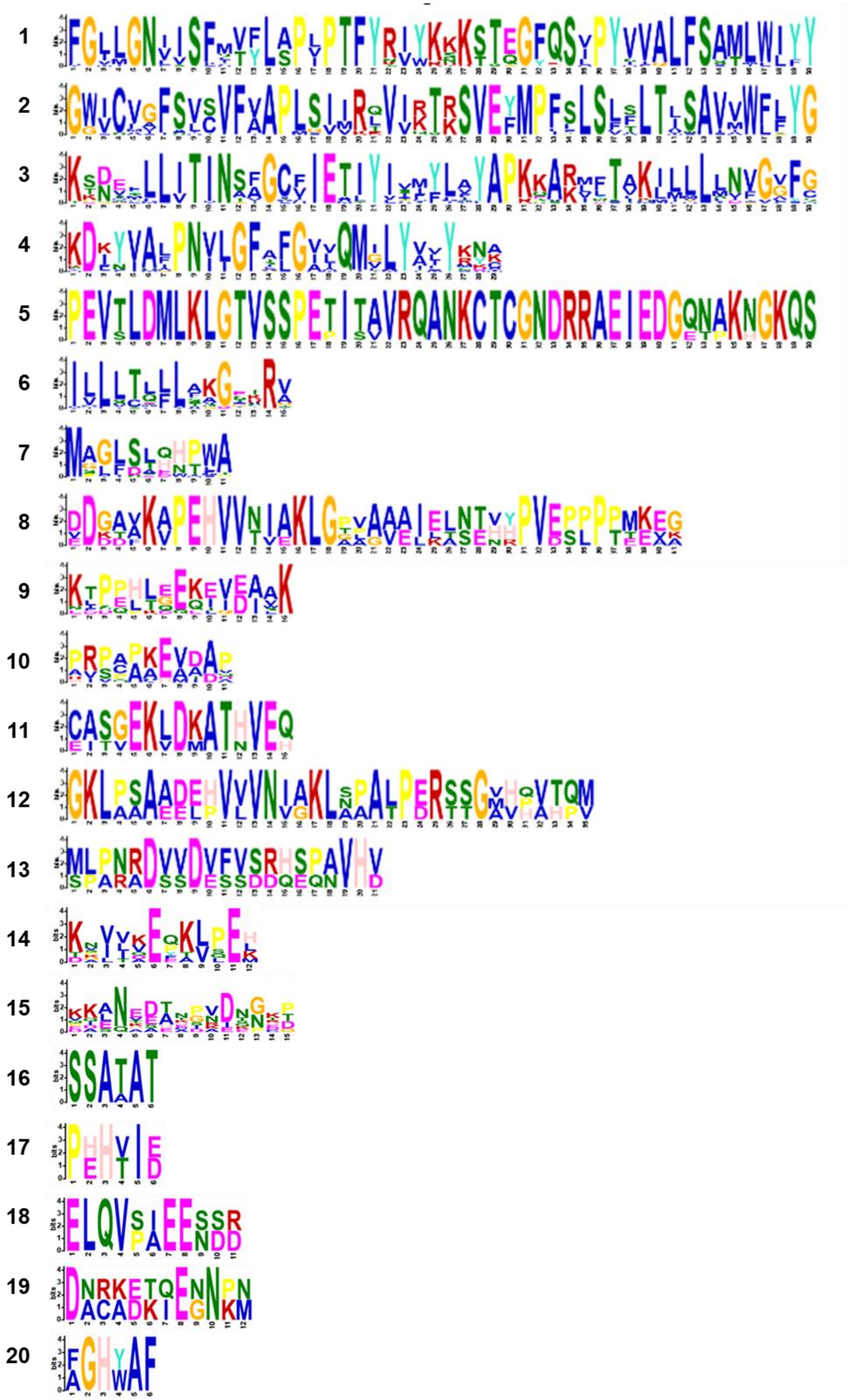

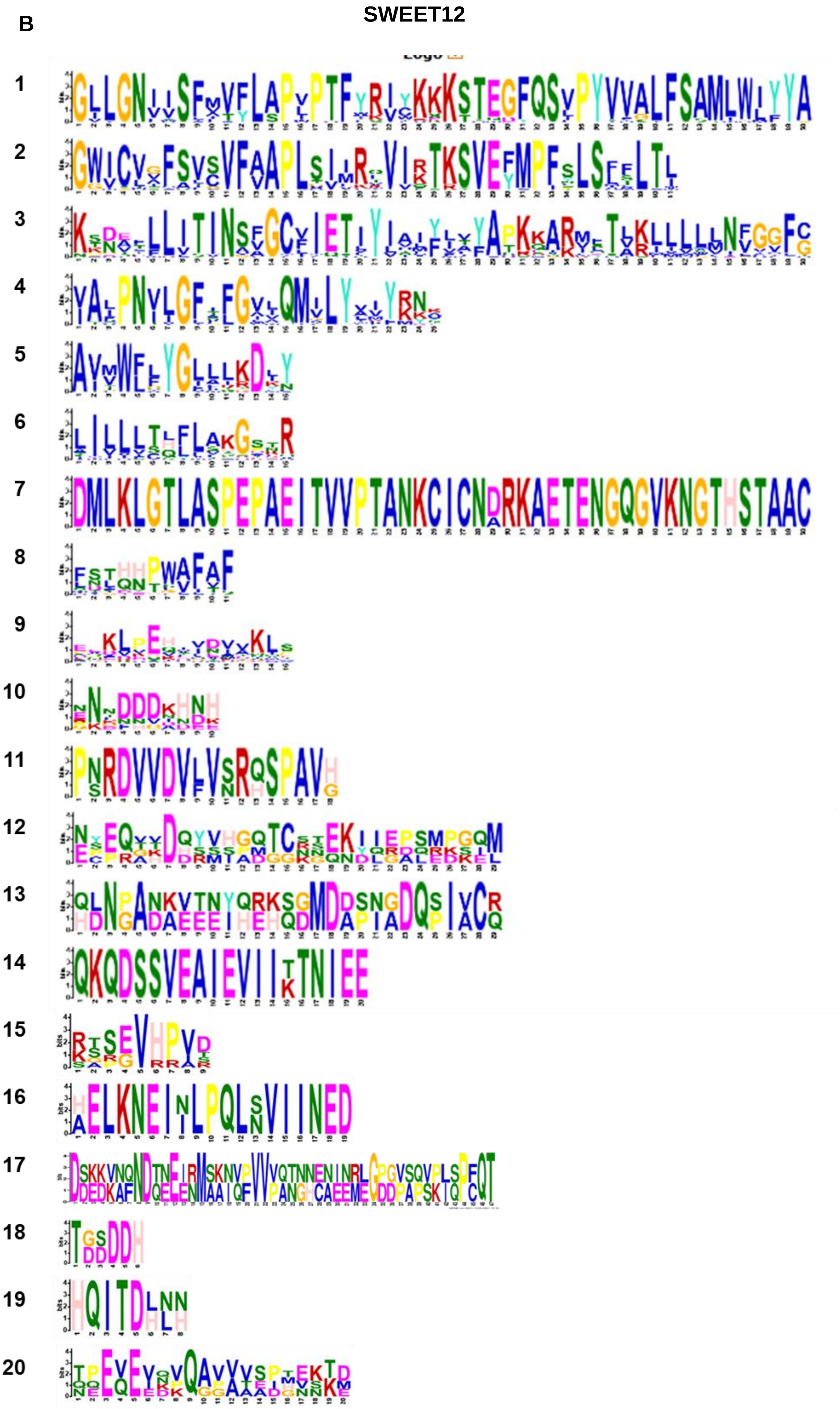
B
SWEET12
1
2
3
4
5
6
7
8
9
10
11
12
13
14
15
16
17
18
19
20

**Supplementary Figure 7. Details of logo of each protein motifs for AtSWEET11 and AtSWEET12 orthologs in different plant species.**

**A and B**, The logo of each motif and associated amino acids were identified for **A,** AtSWEET11 and **B,** AtSWEET12 protein orthologs from different plant species using MEME_suite (https://meme-suite.org/meme/). The amino acid frequencies and level of their conservation were indicated by the heights of letters. The X-axis indicates the length of motif and Y-axis indicates the sequence conservation per site of each letter (i.e., bit score). The numbering of the motifs was done according to its significance, the highly significant motifs i.e. the first four motifs were conserved in most of the sequences used in present study. These motifs corresponds to the transmembrane regions of the proteins, conferring that these orthologous proteins could be performing similar functions.

A

B

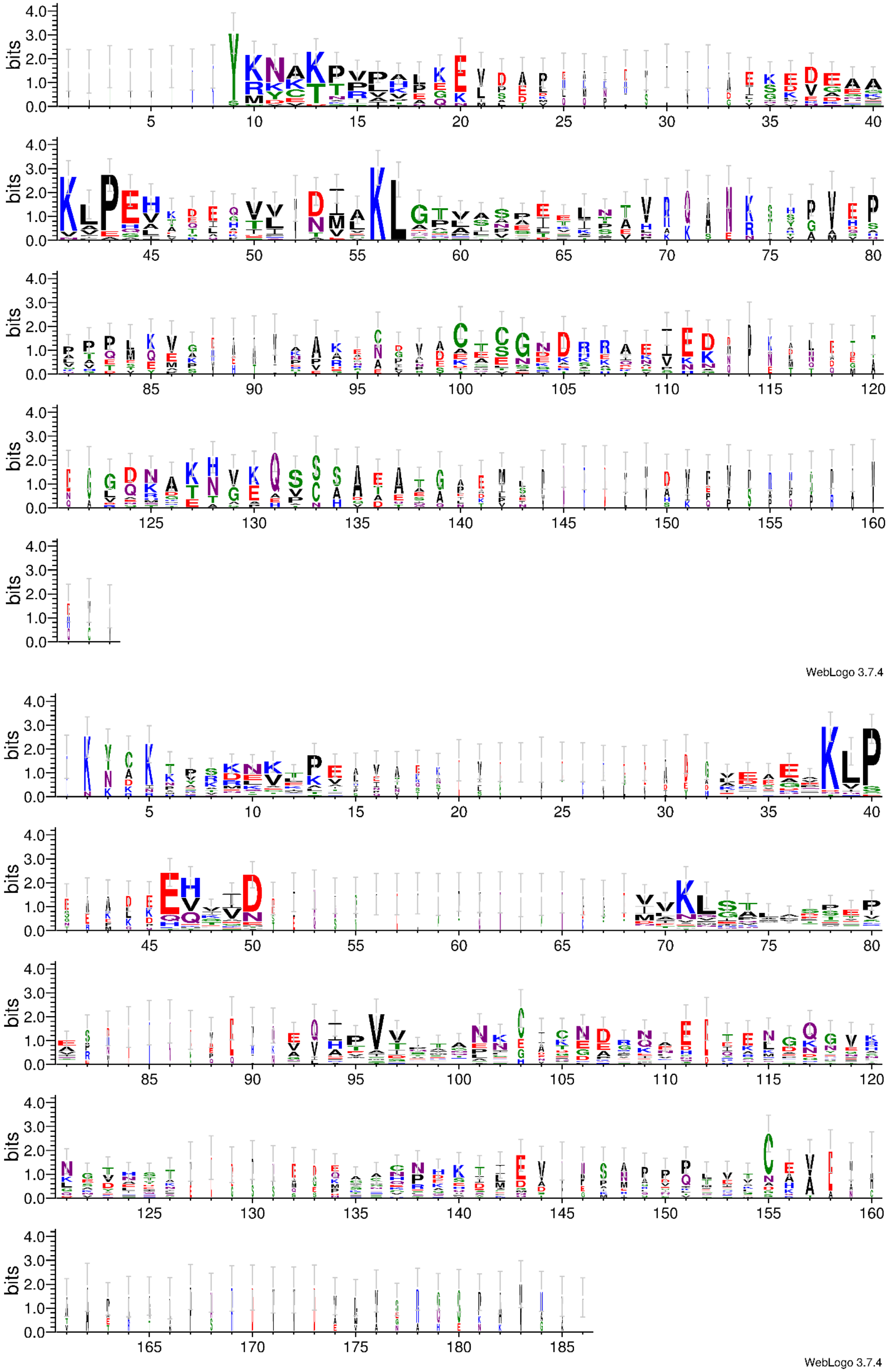

**Supplementary Figure 8. The C-terminal analysis of AtSWEET11 and AtSWEET12 protein orthologs from different plant species.**

The amino acids sequences from C-terminal of **A,** AtSWEET11 and **B,** AtSWEET12 protein orthologs from thirty-nine different plant species were extracted and aligned using the Clustal Omega (https://www.ebi.ac.uk/Tools/msa/clustalo/). The logo of associated amino acids were generated using WebLogo3 (http://weblogo.threeplusone.com/).The amino acid frequencies and level of their conservation were indicated by the heights of letters. The Y-axis indicates the sequence conservation per site of each letter (i.e., bit score). The complete sequence for AtSWEET11 and AtSWEET12 proteins were obtained from ensemble plants/Phytozome. These sequences were used as input for TMHMM to determine the co-ordinates of the C-terminal sequences. Using an in-house perl script, the resulting co-ordinates were then used to extract C-terminal sequences.

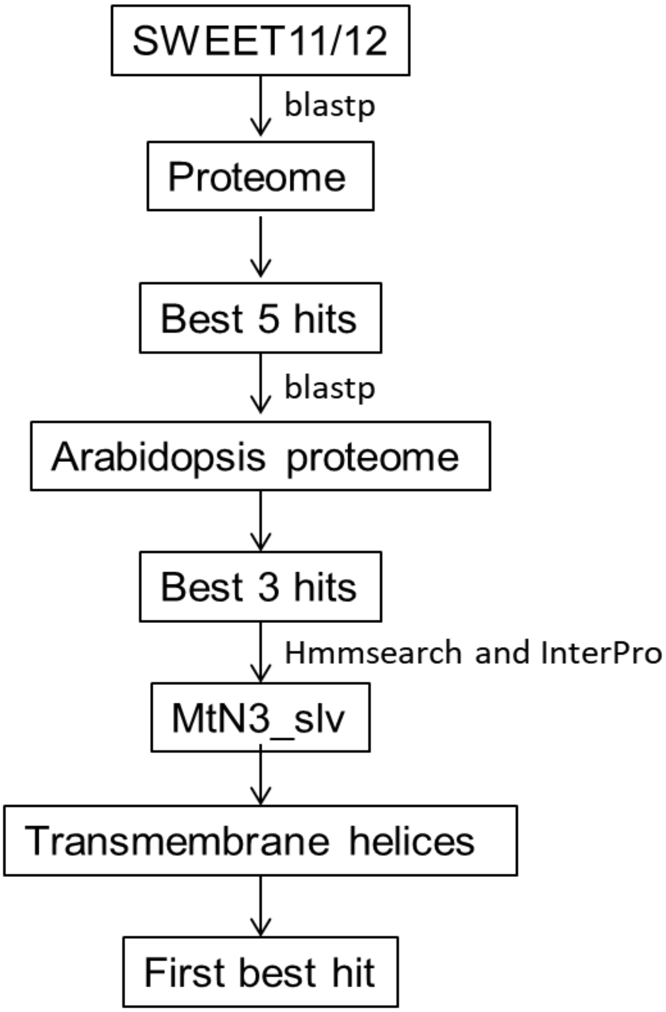


**Supplementary Figure 9. Flowchart for identifying the orthologous genes for AtSWEET11 and AtSWEET12.**
