# Supplementary material for: AtSWEET11 and AtSWEET12 transporters function in tandem to modulate sugar flux in plants
Source: Plant Direct. 2023 Mar 8;7(3):e481. doi: 10.1002/pld3.481 (PMC9995347; doi:10.1002/pld3.481)
Supplement: Supplementary file 2 — Supplementary Table 1: Sucrose‐interacting residues in AtSWEET11 and AtSWEET12 with equivalent dCMP‐binding residues in the AtSWEET13 crystal structure. [file PLD3-7-e481-s003.docx]

**Supplementary Table 1:** **Sucrose interacting residues in AtSWEET11 and AtSWEET12 with equivalent dCMP binding residues in AtSWEET13 crystal structure.**

| AtSWEET11 | AtSWEET12 | AtSWEET13 (ligand binding pocket: PDB: 5XPD) (1) |
| --- | --- | --- |
| S22 | S22 | S20 |
| S56 | S56 | S54* |
| W60 | W60 | W58 |
| N77 | N77 | N76 |
| N197 | N197 | N196 |
| S143 | S143 | S142 |
| S177 | S177 | S176* |
| W181 | W181 | W180 |
| - | - | V23* |
| V146 | V146 | V145* |
| F26 | F26 | - |
| A53 | A53 | - |
| A57 | A57 | - |
| L73 | L73 | - |
| I76 | I76 | - |
| F147 | F147 | - |
| L174 | - | - |
| A178 | - | - |
| P196 | P196 | - |
| G200 | - | - |

* asterisk indicates mutated residues in AtSWEET13-dCMP crystal structure.
