# Supplementary material for: AtSWEET11 and AtSWEET12 transporters function in tandem to modulate sugar flux in plants
Source: Plant Direct. 2023 Mar 8;7(3):e481. doi: 10.1002/pld3.481 (PMC9995347; doi:10.1002/pld3.481)
Supplement: Supplementary file 3 — Supplementary Table 2: Details of conserved residues among AtSWEETs and OsSWEET2b. [file PLD3-7-e481-s004.docx]

**Supplementary Table 2: The details of conserved residues among AtSWEETs and OsSWEET2b.**

| Conserved residues among AtSWEET11 | AtSWEET12 | AtSWEET13 | OsSWEET2b |
| --- | --- | --- | --- |
| G15 | G15 | G13 | G13 |
| G18 | G18 | G16 | G16 |
| N19 | N19 | N17 | N17 |
| P29 | P29 | P27 | P27 |
| T32 | T32 | T30 | T30 |
| P49 | P49 | P47 | P47 |
| Y50 | Y50 | Y48 | Y48 |
| Y63 | Y63 | Y61 | Y61 |
| G136 | G136 | G135 | G136 |
| P150 | P150 | P149 | P150 |
| V157 | V157 | V156 | V157 |
| T160 | T160 | T159 | S160 |
| S162 | S162 | S161 | S162 |
| M166 | M166 | M165 | M166 |
| P167 | P167 | P166 | P167 |
| F168 | F168 | F167 | F168 |
| L170 | L170 | L169 | L170 |
| S171 | S171 | S170 | S171 |
| Y184 | Y184 | Y183 | Y184 |
| D190 | D190 | D189 | D190 |
| N197 | N197 | N196 | N197 |
| Q207 | Q207 | Q206 | Q207 |

The twenty-two conserved residues among AtSWEETs and OsSWEET2b.

T159 (AtSWEET13) is conserved among all AtSWEEts, while S160 is present in OsSWEET2b. N197 is also a part of substrate binding pocket as depicted via AtSWEET13 crystal structure.
